# Predictors of conversion from major depressive disorder to bipolar disorder: A population-based PADRIS-PRESTO cohort study in Catalonia

**DOI:** 10.1192/j.eurpsy.2026.12235

**Published:** 2026-07-02

**Authors:** Meritxell González-Campos, Gerard Anmella, Michele De Prisco, Vincenzo Oliva, Clàudia Valenzuela-Pascual, Ariadna Mas, Marta Korniyenko, Ruth Voltá i Solerdelcoll, Clara Escala, Giovanna Fico, Marc Valentí, Jordi Blanch, Allan H. Young, Eduard Vieta, Diego Hidalgo-Mazzei

**Affiliations:** 1 Departament de Medicina, Facultat de Medicina i Ciències de la Salut, Universitat de Barcelona (UB), Barcelona, Spain; 2 Bipolar and Depressive Disorders Unit, Hospìtal Clinic de Barcelona, Barcelona, Spain; 3 Institut d’Investigacions Biomèdiques August Pi i Sunyer (IDIBAPS), Barcelona, Spain; 4 Institute of Neurosciences (UBNeuro); 5 Centro de Investigación Biomédica en Red de Salud Mental (CIBERSAM), Instituto de Salud Carlos III, Madrid, Spain; 6 Centre for Affective Disorders (CfAD), Institute of Psychiatry, Psychology and Neuroscience (IoPPN), King’s College London, London, UK

**Keywords:** bipolar disorder, diagnostic conversion, electronic health records, major depressive disorder, prediction model

## Abstract

**Background:**

Bipolar disorder (BD) is frequently misdiagnosed as major depressive disorder (MDD), with diagnostic delays averaging 6–10 years leading to suboptimal treatment and poorer outcomes. Early identification of patients at risk for diagnostic conversion remains a critical clinical priority. We aimed to identify clinical predictors of BD conversion in a large population-based cohort and develop a clinically applicable prediction model.

**Methods:**

We conducted a retrospective cohort study using electronic health records from the Catalan PADRIS-PRESTO database (2010–2019). Patients aged ≥18 years with incident MDD diagnosed in specialized mental health services (2015–2019) were followed until BD diagnosis, death, or study end. Time-varying Cox regression addressed proportional hazards violations, with competing risks analysis accounting for mortality.

**Results:**

Among 52,001 patients (median follow-up 4.0 years), 3,958 (7.61%) converted to BD. Psychotic features emerged as the strongest predictor (HR = 3.18, 95% CI: 2.92–3.45, E-value = 5.82), with consistent effects across follow-up. Anxiety disorders showed time-varying protection (baseline HR = 0.41, 95% CI: 0.35–0.48) that attenuated over time (interaction HR = 1.30 per period). The time-varying model achieved good discrimination (C-index = 0.745). Machine learning validation confirmed predictor importance rankings (Spearman *r* = 0.87).

**Conclusions:**

Psychotic features during depressive episodes robustly predict BD conversion, warranting enhanced clinical monitoring. Our time-varying prediction model using routinely collected clinical data enables early risk stratification to guide diagnostic vigilance and treatment decisions.

## Introduction

Bipolar disorder (BD) is a severe mood disorder with a lifetime prevalence of ~1–2% of the world population [1], representing a major cause of disability globally [2, 3]. It typically emerges during adolescence or early adulthood, with a median age of onset around 20 years; early identification is essential to improve prognosis and quality of life [4, 5]. Delayed diagnosis is associated with poorer treatment response, increased suicide risk, and higher healthcare costs [6–10]. Approximately 60% of BD patients initially present with depressive symptoms, often resulting in misdiagnosis as major depressive disorder (MDD) for several years [11]. Diagnostic delays of 6–10 years have been consistently reported [9, 12], during which patients remain on suboptimal or potentially deleterious treatments, particularly antidepressant monotherapy without mood stabilizers, which can trigger manic episodes and worsen outcomes [10, 13, 14].

Multiple research approaches have attempted to identify predictors of BD conversion among MDD patients. Population-based registry studies from Sweden [15], Denmark [16], Taiwan [17], and Korea [18] have identified demographic factors, psychiatric comorbidities, and healthcare utilization patterns as candidate predictors, while electronic health record (EHR) studies using traditional regression [11] and machine learning [14] have achieved moderate predictive accuracy in diverse healthcare systems. Smaller cohorts have also highlighted childhood trauma, substance use, and specific depressive features as potential risk factors [19–21].

Systematic reviews and meta-analyses have synthesized these heterogeneous findings. Kessing et al. examined 31 longitudinal cohorts of patients with unipolar depression and, in 11 cohorts using survival analyses, including register-based samples, reported decreasing annual conversion rates over time without consistent clinical predictors, largely attributed to methodological differences [22]. In contrast, Ratheesh et al. analyzed 56 predominantly prospective clinical MDD cohorts and identified three robust predictors of conversion: family history of BD, younger age of onset, and psychotic features [23]. Salazar de Pablo et al. found that ~15% of children and adolescents with depressive disorders later developed BD, with younger age, recruitment from specialized clinics, and hospitalization associated with increased risk [24]. However, in the EarlyBipolife study, family history alone had limited prognostic value for conversion to BD [25].

Overall, these contributions indicate that BD conversion risk is at least partially predictable, but the available evidence remains heterogeneous. Many predictive models rely on clinical variables incompletely collected in routine practice and sample sizes and follow-up periods vary considerably, limiting generalizability to diverse clinical settings. The Catalan PADRIS-PRESTO program offers a unique opportunity to mitigate some of these limitations. This comprehensive database includes EHRs from the entire public health system of Catalonia. Using this population-based resource, the present study aimed to identify clinical predictors of BD conversion in a large cohort of MDD patients and develop a prediction model applicable to routine clinical practice.

## Methods

### Study design and data source

The PADRIS-PRESTO dataset included 473,812 individuals who accessed public specialized mental health services (outpatient, inpatient, or emergency) between January 1, 2015, and December 31, 2019. Retrospective follow-up extended back to January 1, 2010, using EHR data across the entire period within the Catalan public health system, which serves ~7.5 million inhabitants. Information was anonymized and de-identified in full compliance with legal and ethical standards. Further cohort details are reported elsewhere [26]. The study was approved by the Hospital Clínic de Barcelona Ethics Committee (HCB/2020/0735) and followed the Strengthening the Reporting of Observational Studies in Epidemiology (STROBE) guidelines [27].

### Study population

We identified patients aged ≥18 years with a first recorded diagnosis of MDD (ICD-10 codes F32, F33) in specialized mental health services between 2015 and 2019, requiring at least 1 year of observable healthcare history before diagnosis. For all included patients, we retrieved EHR data from 2010 to 2019 for comprehensive predictor assessment. Patients were excluded if they had any prior diagnosis of BD (F30, F31) or schizophrenia spectrum disorders (F20–F29), or if BD was diagnosed in the same year as MDD. The index date was defined as the first recorded MDD diagnosis. Follow-up extended until BD diagnosis (≥1 year after index), death, loss to follow-up, or study end, whichever occurred first. Loss to follow-up was minimal (<0.1%) due to universal healthcare coverage with comprehensive EHRs.

### Outcome and predictor variables

The primary outcome was diagnostic conversion to BD recorded in specialized mental health services following MDD diagnosis. To reduce misclassifications due to early diagnostic clarification, BD conversion was required to occur at least 1 year after the index MDD diagnosis. Death was treated as a competing risk, as it precludes subsequent BD conversion. All sociodemographic, psychiatric comorbidity, and healthcare utilization variables were assessed during the 1-year lookback window preceding the index MDD diagnosis. Somatic comorbidities were counted as a simple count of distinct nonpsychiatric diagnoses recorded in primary care and hospital discharge data during the 2 years before the index MDD episode, as detailed in Supplementary Methods, rather than using a weighted comorbidity index. Sociodemographic predictors included age at MDD diagnosis, sex, income status, urban versus rural residence, and foreign-born status. Clinical predictors included psychotic features during depressive episodes, anxiety disorders, substance use disorders, personality disorders, number of somatic comorbidities, and suicide attempts. Healthcare utilization predictors included number of primary care visits, mental health outpatient visits, emergency department visits, and psychiatric hospitalizations during the 1-year lookback window.

For the general model (reported in Supplementary Analyses), we additionally included psychotropic medication use in the year before MDD diagnosis, categorized by therapeutic class (antidepressants, antipsychotics, mood stabilizers, and benzodiazepines), as well as the total number of psychotropic classes. Complete operational definitions of variables and coding procedures are provided in Supplementary Methods. The clinical model included 15 predictor variables selected based on clinical relevance, prior evidence on bipolar conversion [15–18, 22–24], and availability in routine clinical practice. Three variables (sex, urban residence, and suicide attempts) were handled as stratification variables in Cox models due to convergence issues, creating separate baseline hazards while maintaining common regression coefficients (detailed in the Supplementary Methods).

### Statistical analysis

Baseline characteristics were compared between converters and nonconverters using standardized mean differences (SMDs).

#### Time-to-event estimation and competing risks

Time-to-event was defined as the interval from the index MDD diagnosis date to the earliest of BD conversion, death, loss to follow-up, or end of study. Because death competes with BD conversion, we estimated the cumulative incidence of BD conversion using the cumulative incidence function (CIF) under competing risks (Aalen–Johansen estimator). Group differences in CIF across predictor strata were assessed using Gray’s test. For descriptive purposes, we also generated Kaplan–Meier curves for cause-specific conversion-free survival, acknowledging that Kaplan–Meier treats competing events as censoring. Conversion-free survival of BD conversion was estimated using Kaplan–Meier methods, with log-rank tests comparing survival curves across predictor strata.

#### Proportional hazards assessment and primary modeling strategy

We assessed the proportional hazards assumption using Schoenfeld residual-based tests, applying Bonferroni correction. Both the clinical and general models demonstrated substantial violations of proportional hazards assumptions, with 60% and 77% of variables showing significant time-varying effects, respectively (Supplementary Table S2). Given the extent of violations, we implemented time-varying coefficient Cox regression as our primary analytical approach. The dataset was split into three clinically meaningful time periods according to follow-up duration: 1–2 years, 2–5 years, and 5+ years, based on the observed distribution of conversion events over time and on typical time windows for diagnostic consolidation in bipolar disorder (early peak, intermediate stabilization, and later residual risk). This structure is consistent with the early peak and subsequent decline in conversion risk observed in our data. The lower bound reflects our ≥1 year follow-up inclusion criterion, while maximum observable follow-up was ~9 years given the 2010–2019 study period. Variables demonstrating time-varying effects were modeled with interactions between the predictor and time period, allowing hazard ratios to differ across follow-up intervals. Full details of proportional hazards testing and time-varying model specification are provided in Supplementary Methods.

#### Model performance: Discrimination and calibration

Model performance was assessed using discrimination and calibration metrics. Discrimination was quantified using Harrell’s concordance index (C-index). Internal validation employed 200 bootstrap resamples to calculate optimism-corrected estimates. We additionally calculated time-dependent area under the ROC curve at 2, 5, and 10 years. Calibration was evaluated by comparing observed versus predicted conversion rates, with agreement tested using the Hosmer–Lemeshow statistic. Detailed validation procedures are described in Supplementary Methods.

#### Competing risks and sensitivity analyses

To account for death as a competing event, we implemented Fine–Gray subdistribution hazard models and compared subdistribution hazard ratios with cause-specific Cox estimates. Six sensitivity analyses were conducted: standard Cox model ignoring time-varying effects, parsimonious model with core clinical predictors only, restriction to patients with at least 2 years of follow-up, analysis restricted to the first 2 years post-diagnosis, restriction to high healthcare utilizers (≥10 mental health visits), and complete case analysis. Full specifications for all sensitivity analyses are provided in Supplementary Methods.

#### Benchmarking against machine learning models

For methodological benchmarking, we compared the time-varying Cox model with machine learning algorithms (LASSO logistic regression, Random Forest, and XGBoost) on identical temporal train-test splits, evaluating discrimination using area under the ROC curve at 5 years. Machine learning methods are described in detail in Supplementary Methods.

All analyses were performed using R version 4.5.1, with a two-sided *α* of 0.05 for statistical significance and no correction for multiple testing of primary model coefficients. Complete specifications of statistical packages, versions, and code availability are provided in Supplementary Methods.

## Results

### Study population

**Figure 1.**
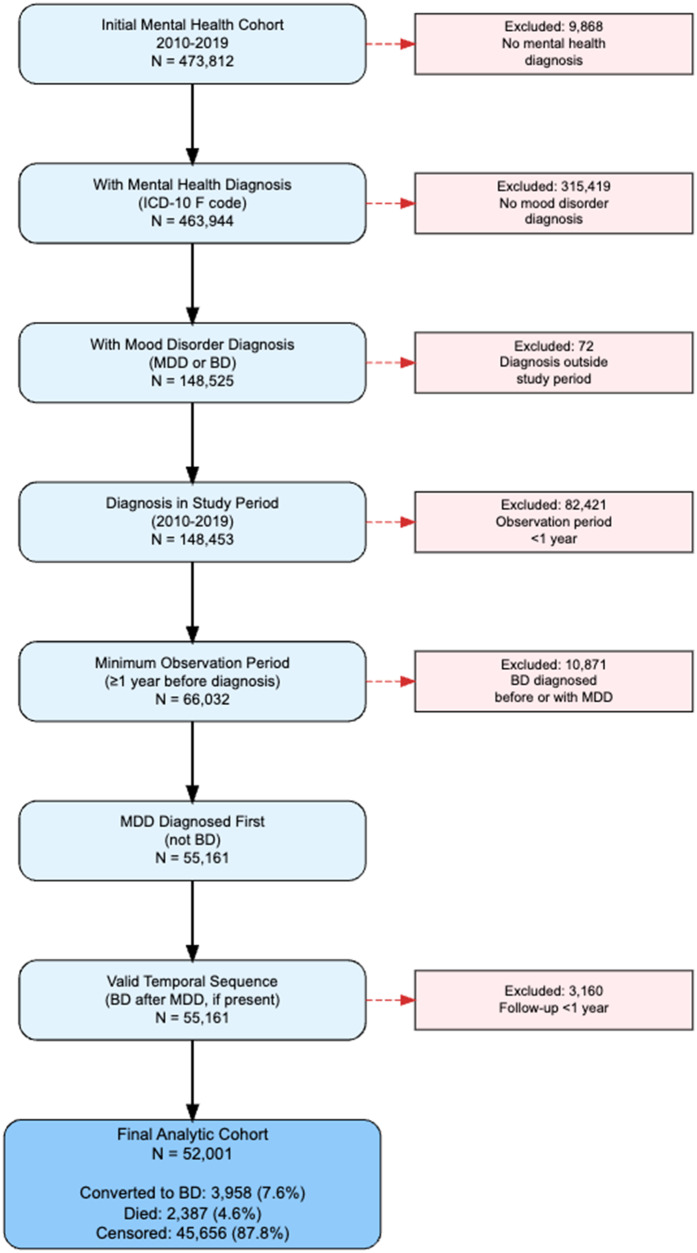
Study flowchart and cohort selection. Flow diagram depicting the cohort selection process for the study of major depressive disorder (MDD) to bipolar disorder (BD) conversion in the PADRIS database, Catalonia, Spain (2010–2019). MDD, major depressive disorder; BD, bipolar disorder; ICD-10, International Classification of Diseases, 10th revision. Figure 1. long description.

From 473,812 individuals with specialized mental health contact between 2015 and 2019, we identified 52,001 patients meeting criteria for incident MDD, with EHR data available from 2010 to 2019. During a median follow-up of 4.0 years (interquartile range: 2.0–5.0), 3,958 patients (7.61%) converted to BD, 45,656 (87.80%) remained with unipolar depression, and 2,387 (4.59%) died (Figure 1). The cumulative incidence of BD conversion was 4.0% at 1 year, 5.8% at 3 years, 8.9% at 5 years (95% CI: 8.6–9.2%), and 14.5% at 10 years, with the highest incidence in the first year post-diagnosis (4.0%), decreasing to ~1.5% annually thereafter.

### Baseline characteristics

Table 1 presents baseline characteristics stratified by conversion status, with 95% confidence intervals and standardized effect sizes. Among the 3,958 converters, median time from MDD diagnosis to BD conversion was 3.0 years (IQR 1.0–5.0, 95% CI: 3.0–3.0). Converters were significantly older at index diagnosis (mean 55.6 years, SD: 15.4, 95% CI: 55.1–56.0 vs. 52.3 years, SD: 17.4, 95% CI: 52.1–52.4; Cohen’s *d* = 0.200).

**Table 1.** Complete baseline characteristics stratified by bipolar disorder conversion status Table 1. long description.

Variable	Total (*N* = 52,001)	Nonconverters (*n* = 48,043)	Converters (*n* = 3,958)	*P*-value	Effect size
**Sociodemographic characteristics**					
Age (years), mean (SD) [95% CI]	52.5 (17.3) [52.4–52.7]	52.3 (17.4) [52.1–52.4]	55.6 (15.4) [55.1–56.0]	<0.001	0.200
Sex				0.027	0.037
Female	33,807 (65.0%) [64.6–65.4]	31,298 (65.1%) [64.7–65.6]	2,509 (63.4%) [61.9–64.9]		
Male	18,194 (35.0%) [34.6–35.4]	16,745 (34.9%) [34.4–35.3]	1,449 (36.6%) [35.1–38.1]		
Annual income level				0.003	0.046
Low (<€18,000/year)	40,923 (78.7%) [78.3–79.0]	37,831 (78.7%) [78.4–79.1]	3,092 (78.1%) [76.8–79.4]		
Middle (€18,001–100,000/year)	10,949 (21.1%) [20.7–21.4]	10,103 (21.0%) [20.7–21.4]	846 (21.4%) [20.1–22.7]		
High (>€100,000/year)	129 (0.2%) [0.2–0.3]	109 (0.2%) [0.2–0.3]	20 (0.5%) [0.3–0.8]		
Residence area				0.057	0.032
Rural	16,210 (31.2%) [30.8–31.6]	15,030 (31.3%) [30.9–31.7]	1,180 (29.8%) [28.4–31.3]		
Urban	35,791 (68.8%) [68.4–69.2]	33,013 (68.7%) [68.3–69.1]	2,778 (70.2%) [68.7–71.6]		
Nationality				0.008	0.046
Spanish	48,994 (94.2%) [94.0–94.4]	45,227 (94.1%) [93.9–94.3]	3,767 (95.2%) [94.5–95.8]		
Non-Spanish	3,007 (5.8%) [5.6–6.0]	2,816 (5.9%) [5.7–6.1]	191 (4.8%) [4.2–5.5]		
Vital status at end of follow-up					
Deceased	2,636 (5.1%) [4.9–5.3]	2,387 (5.0%) [4.8–5.2]	249 (6.3%) [5.6–7.1]	<0.001	0.057
**Clinical characteristics at index episode**					
Psychotic features	2,690 (5.2%) [5.0–5.4]	1,850 (3.9%) [3.7–4.0]	840 (21.2%) [20.0–22.5]	<0.001	0.544
Comorbid substance use disorder	13,863 (26.7%) [26.3–27.0]	12,562 (26.1%) [25.8–26.5]	1,301 (32.9%) [31.4–34.3]	<0.001	0.148
Comorbid anxiety disorder	28,612 (55.0%) [54.6–55.4]	27,075 (56.4%) [55.9–56.8]	1,537 (38.8%) [37.3–40.4]	<0.001	0.356
Comorbid personality disorder	9,586 (18.4%) [18.1–18.8]	8,460 (17.6%) [17.3–18.0]	1,126 (28.4%) [27.1–29.9]	<0.001	0.260
History of suicide attempt	530 (1.0%) [0.9–1.1]	443 (0.9%) [0.8–1.0]	87 (2.2%) [1.8–2.7]	<0.001	0.103
**Healthcare utilization (pre-index period)**					
Mental health hospitalizations, mean (SD) [95% CI]	0.3 (1.2) [0.3–0.3]	0.2 (0.9) [0.2–0.2]	1.1 (2.7) [1.1–1.2]	<0.001	0.456
High illness severity					
No (0–1 hosp.)	48,840 (93.9%) [93.7–94.1]	45,792 (95.3%) [95.1–95.5]	3,048 (77.0%) [75.7–78.3]	<0.001	0.550
Yes (≥2 hosp.)	3,161 (6.1%) [5.9–6.3]	2,251 (4.7%) [4.5–4.9]	910 (23.0%) [21.7–24.3]		
Primary care visits (count), mean (SD) [95% CI]	48.5 (44.9) [48.1–48.9]	48.0 (44.5) [47.6–48.4]	55.3 (49.7) [53.7–56.8]	<0.001	0.155
Mental health outpatient visits, mean (SD) [95% CI]	9.3 (12.9) [9.2–9.4]	8.6 (11.9) [8.5–8.7]	18.4 (19.2) [17.8–19.0]	<0.001	0.618
Emergency department visits, mean (SD) [95% CI]	4.4 (6.9) [4.3–4.4]	4.2 (6.8) [4.2–4.3]	5.8 (8.2) [5.6–6.1]	<0.001	0.208
**Comorbidity burden**					
Number of psychiatric comorbidities, mean (SD) [95% CI]	1.2 (0.8) [1.2–1.2]	1.2 (0.7) [1.2–1.2]	1.4 (1.1) [1.3–1.4]	<0.001	0.132
Number of somatic comorbidities, mean (SD) [95% CI]	0.1 (0.5) [0.1–0.1]	0.1 (0.4) [0.1–0.1]	0.2 (0.6) [0.2–0.2]	<0.001	0.209
**Follow-up duration**					
Follow-up Ttme (years), median (IQR) [95% CI]	4.0 (2.0–5.0) [4.0–4.0]	4.0 (2.0–5.0) [4.0–4.0]	3.0 (1.0–5.0) [3.0–3.0]	<0.001	—

Abbreviations: SMD, standardized mean difference. For continuous variables: Cohen’s d = |mean₁ − mean₂| / pooled SD. For categorical variables: Austin’s standardized difference (Austin, 2009). Values > 0.10 indicate meaningful imbalance.

*Note*: Data presentation: Categorical variables: n (%) [95% CI]; Continuous variables: mean (SD) [95% CI]; Follow-up: median (IQR) [95% CI]. *P*-values: Chi-square test (categorical), t-test (continuous), Wilcoxon test (median).

Clinical severity markers strongly differentiated converters. Psychotic features were present in 21.2% (95% CI: 20.0–22.5) of converters versus 3.9% (95% CI: 3.7–4.0) of nonconverters (Austin’s *d* = 0.544), representing the largest baseline difference. Healthcare utilization patterns revealed substantial disparities: psychiatric hospitalizations averaged 1.1 (SD: 2.7, 95% CI: 1.1–1.2) in converters versus 0.2 (SD: 0.9, 95% CI: 0.2–0.2) in nonconverters (Cohen’s *d* = 0.456), while mental health visits averaged 18.4 (SD: 19.2, 95% CI: 17.8–19.0) versus 8.6 (SD: 11.9, 95% CI: 8.5–8.7), respectively (Cohen’s *d* = 0.618).

Substance use disorders were more prevalent among converters (32.9%, 95% CI: 31.4–34.3 vs. 26.1%, 95% CI: 25.8–26.5; Austin’s *d* = 0.148), whereas anxiety disorders showed an unexpected inverse association (38.8%, 95% CI: 37.3–40.4 vs. 56.4%, 95% CI: 55.9–56.8; Austin’s *d* = 0.356). Personality disorders were substantially more common in converters (28.4%, 95% CI: 27.1–29.9 vs. 17.6%, 95% CI: 17.3–18.0; Austin’s *d* = 0.260). Somatic comorbidity burden was significantly higher in converters (0.2, SD: 0.6, 95% CI: 0.2–0.2 vs. 0.1, SD: 0.4, 95% CI: 0.1–0.1; Cohen’s *d* = 0.209). Complete baseline characteristics with 95% confidence intervals are provided in Table 1.

### Survival analyses

**Figure 2.**
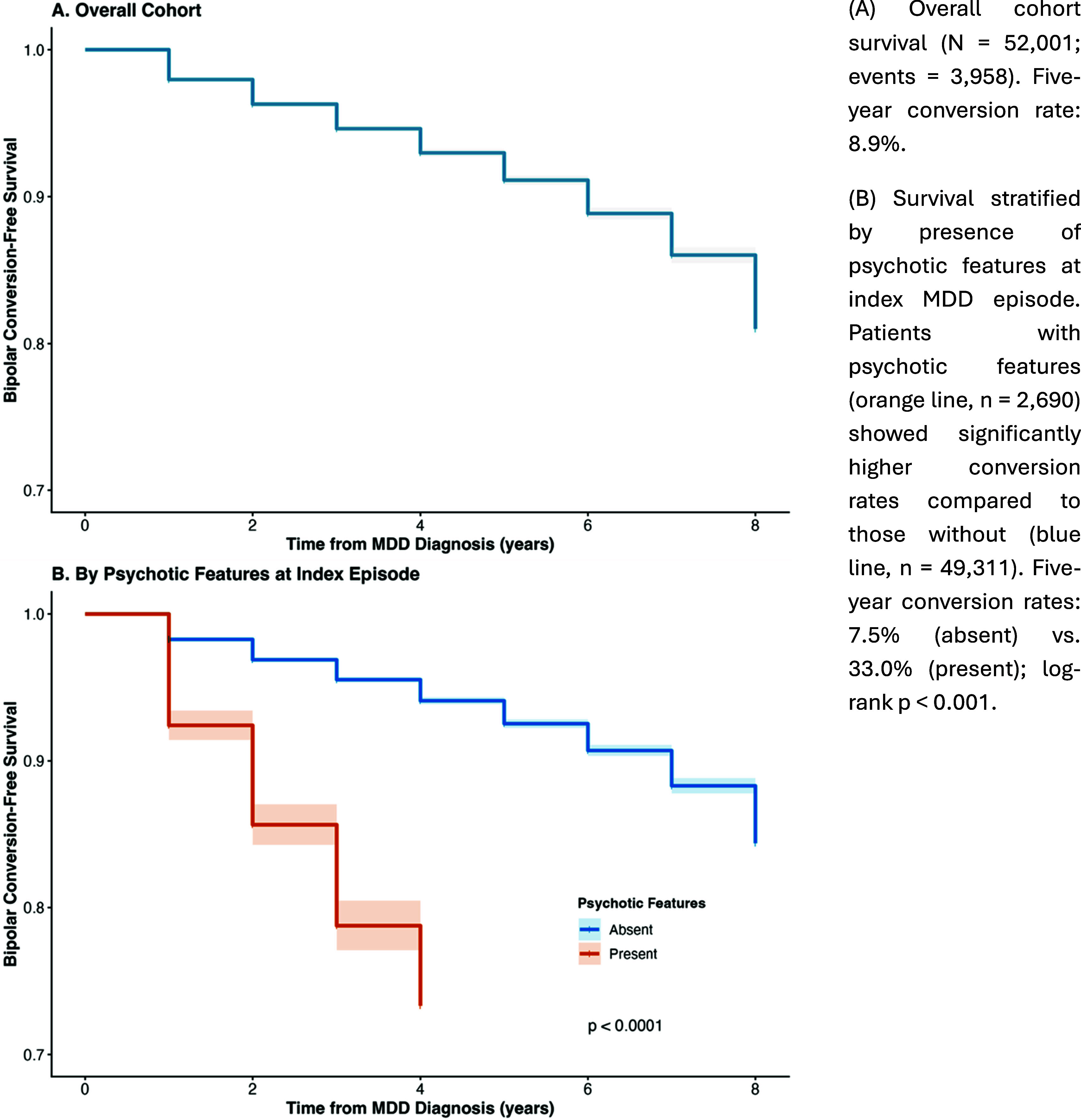
Kaplan–Meier survival curves for diagnostic conversion from major depressive disorder to bipolar disorder in Catalonia, Spain (2010–2019). (A) Overall cohort survival (*N* = 52,001; events = 3,958). Five-year conversion rate: 8.9%. (B) Survival stratified by presence of psychotic features at index MDD episode. Patients with psychotic features (orange line, *n* = 2,690) showed significantly higher conversion rates compared to those without (blue line, *n* = 49,311). Five-year conversion rates: 7.5% (absent) versus 33.0% (present); log-rank *p* < 0.001. Figure 2. long description.

Kaplan–Meier curves demonstrated distinct conversion trajectories across clinical subgroups (Figure 2). Overall conversion-free survival at 5 and 10 years was 91.1% (95% CI: 90.8–91.4%) and 85.5%, respectively (Figure 2A). Stratification by psychotic features revealed substantial risk differentiation (log-rank *p* < 0.001, Figure 2B). Patients without psychotic features maintained 92.5% conversion-free survival at 5 years, whereas those with psychotic features showed 67.0%, corresponding to a 3.8-fold higher conversion risk and underscoring psychotic features as a critical early risk indicator.

### Model selection process

Proportional hazards testing revealed extensive violations in 9 of 15 predictors (60%) in the clinical model (global Schoenfeld test *χ*^2^ = 680.25, *p* < 0.001), indicating that predictor effects change over time and invalidating standard Cox assumptions (Supplementary Table S2). These violations necessitated advanced modeling approaches capable of capturing temporal dynamics in risk associations. We, therefore, developed two parallel modeling strategies distinguished by predictor sets: clinical models utilized only prediagnosis patient characteristics and healthcare utilization patterns, whereas general models additionally incorporated medication variables. Although general models achieved higher discrimination (*C* = 0.874 vs. 0.745), we present clinical models as the primary analyses due to methodological concerns. Medication variables were excluded from primary analyses due to circularity bias, as prescribing patterns likely reflect diagnostic suspicion rather than independent risk factors (detailed in Methods). General model results, including medications, are presented in the Supplementary Results Section B and Supplementary Table S5.

### Primary time-varying cox regression

To address proportional hazards violations while preserving interpretability, we implemented a time-varying coefficient Cox model dividing follow-up into three clinically relevant periods (1–2, 2–5, and 5+ years). This approach directly estimates how predictor effects evolve over time. The clinical time-varying model achieved a concordance index of 0.745 (SE = 0.005), representing a 11.3% improvement over standard Cox regression that incorrectly assumes constant hazards (*C* = 0.669, *p* < 0.001). Table 2 presents baseline hazard ratios (Period 1: 1–2 years) and time interaction effects for all predictors. Time-invariant predictors maintained consistent associations throughout follow-up. Age demonstrated a stable effect (HR = 1.009 per year, 95% CI: 1.007–1.011, *p* < 0.001), translating to a 9% increased risk per decade. Psychotic features emerged as the strongest predictor with remarkably stable effects across all periods (baseline HR = 3.18, 95% CI: 2.92–3.45, *p* < 0.001; time interaction HR = 1.01, 95% CI: 0.94–1.08, *p* = 0.341), tripling conversion risk consistently.

**Table 2.** Time-varying cox regression model results for clinical predictors of bipolar disorder conversion Table 2. long description.

	Variable	Baseline HR (1–2 years)	95% CI	Time interaction HR	95% CI	*E*-value (baseline)	*P*-value
Time-invariant predictors	Age (per year)	1.009	(1.007–1.011)	—	—	1.10	<0.001
	Low income	0.805	(0.745–0.869)	—	—	1.79	<0.001
	Foreign born	1.049	(0.905–1.217)	—	—	1.28	0.524
	Psychotic features	3.183	(2.929–3.458)	—	—	5.82	<0.001
	Personality disorder	1.206	(1.118–1.301)	—	—	1.70	<0.001
Time-varying predictors	Somatic comorbidities	1.259	(1.082–1.464)	0.990	(0.923–1.062)	1.83	0.003
	Substance use disorders	0.947	(0.796–1.127)	1.180	(1.077–1.294)	1.30	0.539
	Anxiety disorders	0.407	(0.344–0.481)	1.306	(1.196–1.427)	4.35	<0.001
	Primary care visits	0.985	(0.983–0.988)	1.008	(1.007–1.008)	1.14	<0.001
	Mental health visits	1.013	(1.009–1.018)	1.002	(1.000–1.004)	1.13	<0.001
	MH hospitalizations	1.141	(1.118–1.165)	0.937	(0.924–0.950)	1.54	<0.001
	Emergency visits	1.004	(0.995–1.014)	1.000	(0.995–1.005)	1.07	0.376

Abbreviations: HR, hazard ratio; CI, confidence interval; MH, mental health.

*Note:* Baseline hazard ratios represent Period 1 (1–2 years). Time interaction indicates change in hazard ratio per subsequent period. Time periods: Period 1 (1–2 years), Period 2 (2–5 years), Period 3 (+5 years). Note: The ≥1 year follow-up inclusion criterion precludes events before year 1, and maximum follow-up of ~9 years (given the 2010–2019 study period) limits extended observations. E-values represent the minimum strength of association that an unmeasured confounder would need to have with both the predictor and outcome to fully explain away the observed effect. Variables used for stratification in the original Cox model (sex, urban residence, and suicide attempts) are not included in the time-varying model.

**Figure 3.**
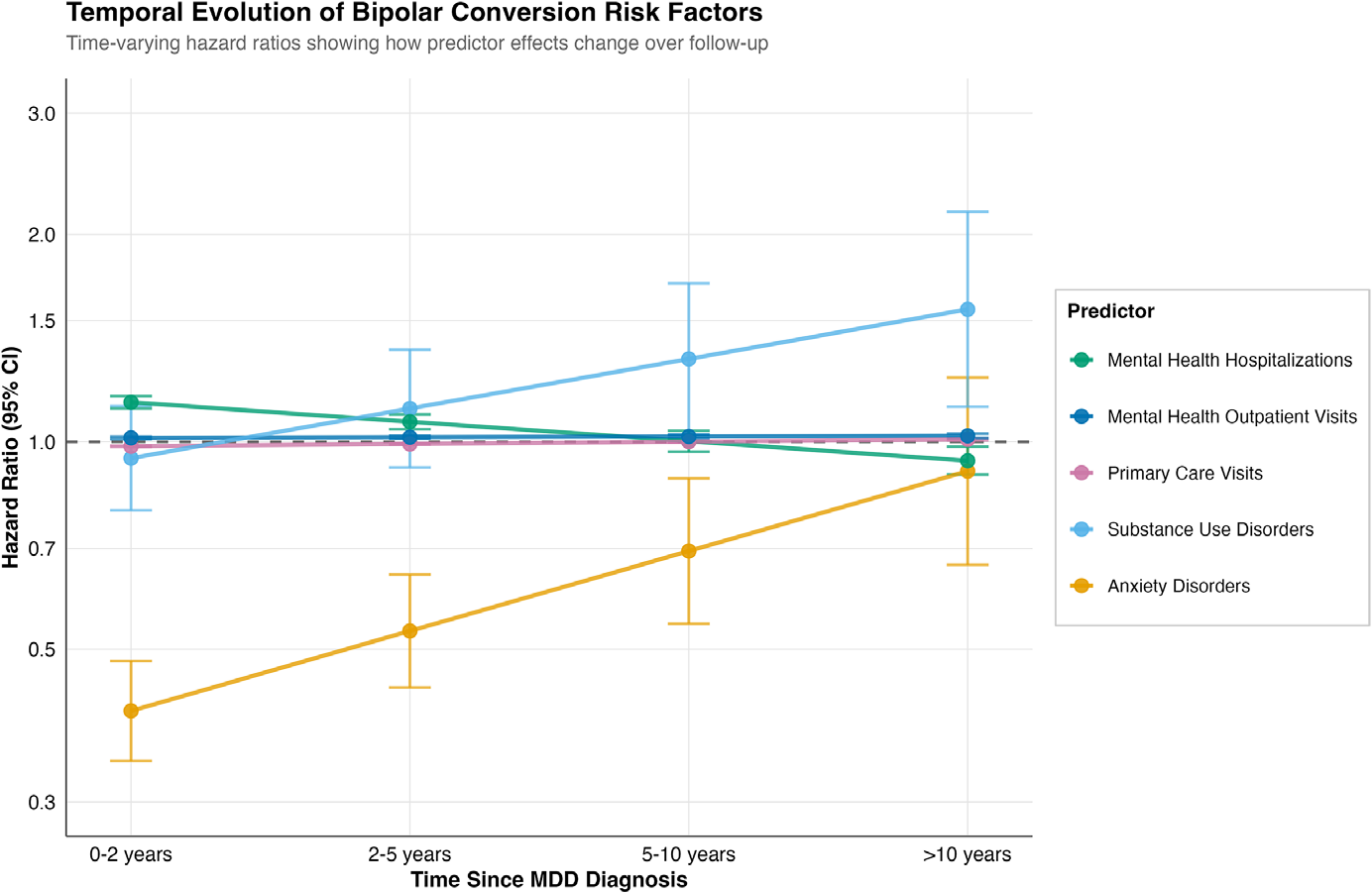
Temporal evolution of bipolar conversion risk factors. Time-varying hazard ratios showing how predictor effects change across three follow-up periods (1–2 years, 2–5 years, and 5+ years) since initial major depressive disorder diagnosis. Points represent hazard ratio estimates with error bars indicating 95% confidence intervals. The dashed horizontal line indicates HR = 1.0 (no effect). Anxiety disorders (orange) showed initial protective effects (HR < 1) that attenuated over time, while substance use disorders (blue) demonstrated increasing risk over extended follow-up. Mental health hospitalizations (green) showed diminishing predictive value over time. Primary care visits (pink) and mental health outpatient visits (dark blue) maintained relatively stable effects across follow-up periods. Time periods derived from time-varying Cox regression with piecewise constant hazards. Figure 3. long description.

Time-varying predictors revealed complex temporal dynamics (Figure 3). Anxiety disorders showed marked early protection (baseline HR = 0.41, 95% CI: 0.35–0.48, *p* < 0.001) that progressively attenuated (time interaction HR = 1.30 per period, 95% CI: 1.19–1.42, *p* < 0.001), with HR evolving from 0.41 (1–2 years) through 0.53 (2–5 years) to 0.69 (+5 years) approaching neutrality in extended follow-up. Substance use disorders showed the opposite pattern, with nonsignificant baseline effects (HR = 0.95, 95% CI: 0.80–1.13, *p* = 0.561) but significant positive time interaction (HR = 1.19 per period, 95% CI: 1.09–1.30, *p* < 0.001). Risk emerged progressively: HR = 0.95 (1–2 years), 1.13 (2–5 years), and 1.35 (5+ years). Mental health hospitalizations demonstrated a strong initial association (baseline HR = 1.14 per hospitalization, 95% CI: 1.12–1.17, *p* < 0.001) that attenuated over time (time interaction HR = 0.94, 95% CI: 0.92–0.95, *p* < 0.001). Complete time-varying coefficients for all predictors are presented in Supplementary Table S1, with additional temporal evolution patterns in Supplementary Figure S1.

### Robustness to unmeasured confounding

E-value analysis quantified robustness to unmeasured confounding (Supplementary Table S3). For the critical early period (1–2 years), psychotic features demonstrated exceptional robustness (E-value = 5.82, lower CI: 5.29), exceeding the strength of any known confounder in psychiatric epidemiology. The protective effect of anxiety disorders (E-value = 4.35, lower CI: 2.97) also appeared unlikely to be explained solely by unmeasured confounding. These values support that key associations reflect true relationships rather than confounding artifacts.

### Competing risks analysis

Competing risks analysis accounting for death (*n* = 2,387, 4.59%) using Fine–Gray subdistribution hazard models produced subdistribution hazard ratios within 8% of cause-specific estimates for all predictors. The largest difference occurred for somatic comorbidities (cause-specific HR = 1.27 vs. subdistribution HR = 1.17), reflecting their association with mortality, whereas psychotic features and anxiety disorders showed minimal change (<3%), indicating that these associations are not confounded by differential mortality. Complete competing risks results are presented in Supplementary Results Section A.

### Model performance and validation

The clinical time-varying model demonstrated excellent discrimination (*C* = 0.745, SE = 0.005) with minimal optimism on bootstrap internal validation (optimism-corrected *C* = 0.720, 95% CI: 0.706–0.733). Temporal validation showed negligible performance degradation (training cohort 2010–2015: *C* = 0.747; validation cohort 2016–2019: *C* = 0.741, 0.8% relative decline), confirming robust generalizability despite evolving diagnostic practices. Complete validation metrics, including bootstrap results, temporal validation, and time-dependent AUC, are provided in Supplementary Table S4 and Supplementary Results Section A.

### Sensitivity analyses

Sensitivity analyses confirmed robustness of findings (Supplementary Results Section A). A standard Cox model that ignored time-varying effects showed lower discrimination (*C* = 0.669 vs. 0.745, *p* < 0.001) and attenuated hazard ratios, whereas a parsimonious model with four core predictors (age, psychotic features, anxiety disorders, and hospitalizations) maintained good performance (*C* = 0.692). Additional analyses addressing immortal time bias, missing data, restriction to early conversions, and high healthcare utilization produced similar effect estimates.

### Machine learning validation and convergence

To validate findings using alternative methodologies, we compared the time-varying Cox approach with LASSO logistic regression, Random Forest, and XGBoost using identical clinical predictors and temporal data splitting. Machine learning models achieved comparable discrimination (XGBoost AUC = 0.783, Random Forest AUC = 0.768, LASSO AUC = 0.763) to the Cox model (*C* = 0.745). All approaches consistently highlighted psychotic features, mental health hospitalizations, age, and somatic comorbidities as top predictors. The correlation between XGBoost feature importance and Cox hazard ratios was 0.87 (Spearman *r*, *p* < 0.001), providing strong convergent validity across different modeling approaches. Full machine learning results are shown in Supplementary Figure S3 and Supplementary Results Section A.

## Discussion

This population-based study of 52,001 patients with incident MDD identified psychotic features as the strongest predictor of BD conversion, with psychiatric hospitalizations showing significant time-varying effects. Overall, 7.61% of patients converted to BD during a median follow-up of 4 years, with the highest incidence in the first year (4%) and ~1.5% annually thereafter, closely replicating findings from large-scale registry studies.

Our conversion rate aligns with contemporary registry studies, which report cumulative incidences around 6–9% and similarly elevated risk in the first years after initial depression diagnosis, supporting the stability of conversion rates across Western European healthcare systems [15]. The shorter median interval from MDD diagnosis to BD conversion in our cohort reflects measurement from formal diagnosis in specialized care with active surveillance, yet still represents clinically meaningful exposure to potentially suboptimal treatment and underscores the value of early risk stratification.

Psychotic features emerged as the strongest predictor with exceptional robustness to unmeasured confounding and showed convergent validity across independent studies and meta-analyses, highlighting psychosis as a core risk marker [16]. Healthcare utilization intensity also showed time-varying effects, with psychiatric hospitalizations strongly associated with conversion early on and attenuating over time, consistent with prior registries identifying inpatient treatment as a strong predictor [15] .

Our finding that older age slightly increased conversion risk diverges from most prior work, which generally reports highest risk in younger adults [18, 23]. This discrepancy may reflect differences in cohort composition, diagnostic practices, and healthcare access rather than a universal age–risk relationship; for example, Miola et al. reported that greater cumulative antidepressant exposure was associated with later age at onset of first-episode mania, which may indicate an influence of age over cumulative antidepressant use and conversion risk [28].

The time-varying anxiety effects, with apparent early protection that attenuates over time, represent a novel finding. Prior studies have shown mixed associations, and unexamined temporal dynamics may partly explain these inconsistencies. This pattern is unlikely to reflect a truly causal protection of anxiety against bipolar disorder and more plausibly reflects diagnostic and coding processes: anxious depressions may be preferentially conceptualized and managed as MDD plus anxiety, whereas “non-anxious” severe or atypical depressions may more often reflect emerging bipolar disorder and thus be re-labeled as such during follow-up. This interpretation is also consistent with the gradual attenuation of the apparent protective effect over time in our time-varying analyses.

The higher prevalence of substance use disorders among converters in our cohort is consistent with evidence that comorbid substance use is closely linked to bipolar onset and worse clinical outcomes. These observations reinforce the importance of systematically assessing and addressing substance use in patients with major depressive disorder who may be at risk for future bipolar conversion. Moreover, although our data do not allow us to disentangle specific substances, recent longitudinal work has suggested that adolescent cannabis use may play a contributory role in triggering bipolar disorder or manic/hypomanic episodes within a broader multicausal vulnerability framework [29].

In time-varying analyses, substance use disorders showed little association with conversion risk in the first 2 years but increasingly elevated hazard ratios thereafter, indicating that SUDs may become more salient markers of bipolar trajectories as the course unfolds. This delayed emergence is consistent with the possibility that persistent or escalating substance use either reflects or contributes to more complex depressive courses in which bipolar disorder is diagnosed only later, and therefore does not necessarily contradict prior reports identifying SUDs as an early risk factor but suggests that their prognostic relevance extends beyond the early course.

The absence of family psychiatric history represents our most significant limitation, as multiple studies identify family history as the strongest predictor [16]. Our model’s C-index of 0.745 despite this limitation suggests that clinical variables capture substantial predictive information, but incorporating family history would likely improve discrimination meaningfully.

Our model performance compares favorably with diverse approaches, including regularized logistic regression [14], classification trees [17], and elastic net models developed in other health systems, which achieve similar discrimination (AUC or C-index around 0.72–0.78) [11]. Within our cohort, machine learning methods achieved only marginally better discrimination, and feature importance analyses consistently highlighted psychotic features, hospitalizations, and mental health visits as top predictors across methods, supporting the robustness of these risk factors. The consistency of performance around C-indices of 0.74–0.78 across methodologies and populations suggests a performance ceiling with current variables, with substantial improvements requiring family history or genetic risk scores. Our machine-learning benchmarking analyses showed that XGBoost, Random Forest, and LASSO models achieved discrimination that was only modestly higher than the time-varying Cox model, while converging on the same core predictors, particularly psychotic features, younger age, and markers of high psychiatric service use. Taken together, these benchmarking results illustrate that artificial intelligence can add value to clinical risk modeling without replacing traditional statistical approaches. In line with recent debates on artificial intelligence in bipolar disorder, including Bartoli et al. [30], machine-learning methods may further enhance clinical risk modeling by integrating large, heterogeneous data sources, capturing nonlinear relationships and interactions between predictors, and supporting the incorporation of risk estimates into digital decision-support tools, both to improve early diagnostic differentiation and to provide individualized predictions of conversion risk over time.

Our findings suggest several potential applications requiring prospective validation. Patients with psychotic features, whose risk is tripled, might warrant cautious antidepressant use and consideration of mood-stabilizing agents, a practice that some clinicians might have already adopted by serendipity [31]. High-risk patients could also receive enhanced early intervention about bipolar warning signs, potentially enabling earlier recognition.

Pharmacological treatment is also likely to interact with conversion risk: different antidepressant classes and specific agents, combination or add-on strategies such as adding mood stabilizers or atypical antipsychotics to antidepressants, and early use of mood stabilizers may influence both the probability and timing of bipolar diagnosis in routine practice. Prior literature suggests that some antidepressants, particularly when used without adequate mood-stabilizing coverage, can precipitate hypomanic or manic switches in vulnerable individuals, whereas early prescription of mood stabilizers or atypical antipsychotics often reflects clinicians’ emerging suspicion of bipolar spectrum disorder. In our cohort, however, medications were only included in a separate general model that improved discrimination but is inherently affected by indication and circularity, so medication variables should be interpreted as markers of clinical complexity and evolving diagnostic formulations rather than as class- or agent-specific causal effects.

### Limitations

Important limitations of our work include the absence of family psychiatric history, the strongest predictor in prior studies [15, 16], although this limitation is shared by most registry studies [14, 17], whereas clinical cohorts with family history often have smaller samples and selection bias [21]. The reliance on ICD-10 diagnostic codes may introduce some misclassification, though this affects all registry studies and is partly offset by ecological validity and the highly replicable findings from Danish and Swedish registers [15]. Restriction to specialized mental health services excludes primary care depression, yet enhances diagnostic validity and targets the population in which BD conversion risk is most clinically relevant. The lack of detailed symptom phenotyping reflects an inherent trade-off in large population-based cohorts, where such assessments are infeasible at scale.

In particular, some depressive phenotypes that have been historically linked to the bipolar spectrum, especially atypical and mixed depressive presentations, could not be evaluated because they are not systematically coded in PADRIS. Clinical studies have shown that atypical depression is closely associated with bipolar II and bipolar-spectrum disorders and may act as a bridge phenotype between unipolar and bipolar II depression [32]. More recent reviews also emphasize that recurrent, often atypical, depressive episodes dominate the longitudinal course of bipolar II disorder and contribute to its frequent misclassification as unipolar major depression [33]. Because ICD-10 diagnoses in our dataset do not reliably capture atypical or mixed specifiers, our models cannot address the prognostic role of these features, and their contribution to bipolar conversion risk is therefore likely to be underestimated. Finally, although generalizability beyond the Catalan healthcare context requires caution, the consistency of our results with Scandinavian registries [15, 16] and convergent findings across continents [14, 17, 21] support broader applicability of these predictors.

Additionally, the inherent nature of EHR-derived data presents intrinsic limitations warranting consideration. The retrospective data collection extending to 2010 precluded definitive exclusion of MDD diagnoses before the database start date; consequently, some patients may have had earlier depressive episodes not captured in our cohort definition. Furthermore, certain sociodemographic and clinical variables such as comorbidities were assessed cumulatively across the study period rather than at specific time points relative to the index MDD diagnosis. Although we restricted predictor assessment to the 1-year period preceding the index date to ensure temporal precedence, this aggregation approach may not fully capture the temporal sequence of comorbidity development relative to mood disorder onset. These limitations are common to population-based registry studies but should be weighed against the ecological validity of findings reflecting real-world clinical practice.

Despite these limitations, major strengths of our study include population-based ascertainment covering all specialized mental health services for 7.5 million inhabitants, minimizing selection bias; a large sample size (52,001 patients, 247,841 person-years) enabling precise estimation of conversion risks and predictor effects; and methodological innovations such as time-varying coefficient Cox regression revealing temporal risk dynamics, explicit management of medication circularity bias, and E-value analysis quantifying robustness to unmeasured confounding. Comprehensive validation, including bootstrap procedures, temporal validation across independent time periods, competing risks analysis, machine learning benchmarking, and multiple sensitivity analyses, further supports the robustness of our findings.

## Conclusion

This study demonstrates that clinically available variables at MDD diagnosis enable meaningful BD conversion prediction, with psychotic features and high illness severity emerging as the strongest predictors showing exceptional robustness to unmeasured confounding. Time-varying Cox regression accounting for temporal risk dynamics achieved moderate discrimination. The consistency of findings with methodologically diverse studies across multiple healthcare systems provides robust evidence for identified risk factors.

Our findings could be complemented through multiple research avenues. Novel biomarker integration offers immediate improvement potential, with RNA editing-based blood tests achieving 82.5% discrimination accuracy [34], and inflammatory cytokine panels showing moderate performance [35, 36] Genetic risk stratification through polygenic risk scores combined with clinical predictors could meaningfully improve discrimination [37, 38], while neuroimaging approaches have identified structural and functional connectivity patterns distinguishing future converters in familial high-risk samples [39, 40].

Most critically, clinical utility remains unproven. Prospective randomized trials comparing risk-adapted interventions are essential to demonstrate whether prediction translates to improved outcomes. Future research should prioritize systematic family history collection, external validation across diverse healthcare systems, and mechanistic studies examining conversion neurobiology to transform statistical prediction into clinical value.

## Supporting information

10.1192/j.eurpsy.2026.12235.sm001 González-Campos et al. supplementary material González-Campos et al. supplementary material

## Supplementary material

The supplementary material for this article can be found at http://doi.org/10.1192/j.eurpsy.2026.12235.

## Long descriptions

### Figure 1 Long description

The flowchart consists of eight central blue boxes connected by downward black arrows, with six red exclusion boxes branching to the right via dashed red arrows.

1. Initial Mental Health Cohort 2010-2019, N = 473,812. Branch right: Excluded 9,868 with no mental health diagnosis.

2. With Mental Health Diagnosis I C D-10 F code, N = 463,944. Branch right: Excluded 315,419 with no mood disorder diagnosis.

3. With Mood Disorder Diagnosis M D D or B D, N = 148,525. Branch right: Excluded 72 with diagnosis outside study period.

4. Diagnosis in Study Period 2010-2019, N = 148,453. Branch right: Excluded 82,421 with observation period less than 1 year.

5. Minimum Observation Period greater than or equal to 1 year before diagnosis, N = 66,032. Branch right: Excluded 10,871 where B D was diagnosed before or with M D D.

6. M D D Diagnosed First not B D, N = 55,161.

7. Valid Temporal Sequence B D after M D D, if present, N = 55,161. Branch right: Excluded 3,160 with follow-up less than 1 year.

8. Final Analytic Cohort, N = 52,001. This final box includes three outcomes: Converted to B D: 3,958 (7.6 percent), Died: 2,387 (4.6 percent), and Censored: 45,656 (87.8 percent).

Navigate back to Figure 1.

### Table 1 Long description

The table is organized into six columns: Variable, Total (N = 52,001), Nonconverters (n = 48,043), Converters (n = 3,958), P-value, and Effect size.

Sociodemographic characteristics:

* Age: Converters are older (mean 55.6) than nonconverters (mean 52.3), P < 0.001.

* Sex: Females represent 65.0% of the total population.

* Annual income: Most participants (78.7%) earn less than 18,000 Euros per year.

* Residence: 68.8% live in urban areas.

* Nationality: 94.2% are Spanish.

Clinical characteristics at index episode:

* Psychotic features: Significantly higher in converters (21.2%) compared to nonconverters (3.9%), P < 0.001, effect size 0.544.

* Comorbidities: Converters show higher rates of substance use disorder (32.9% vs 26.1%) and personality disorders (28.4% vs 17.6%), but lower rates of anxiety disorders (38.8% vs 56.4%).

* Suicide attempt history: Higher in converters (2.2% vs 0.9%).

Healthcare utilization (pre-index period):

* Mental health hospitalizations: Converters had a higher mean (1.1) than nonconverters (0.2).

* High illness severity (2 or more hospitalizations): 23.0% of converters vs 4.7% of nonconverters.

* Outpatient visits: Converters averaged 18.4 visits compared to 8.6 for nonconverters.

Comorbidity burden and follow-up:

* Psychiatric comorbidities: Mean of 1.4 for converters and 1.2 for nonconverters.

* Follow-up time: Median of 3.0 years for converters and 4.0 years for nonconverters.

Navigate back to Table 1.

### Figure 2 Long description

The figure consists of two panels, A and B. Both share an x-axis titled Time from M D D Diagnosis (years) ranging from 0 to 8, and a y-axis titled Bipolar Conversion-Free Survival ranging from 0.7 to 1.0.

* Panel A, titled Overall Cohort, displays a single blue step-down line. The curve begins at 1.0 and descends in annual increments, reaching approximately 0.85 at year 8.

* Panel B, titled By Psychotic Features at Index Episode, compares two groups. The blue line represents Absent psychotic features and follows a gradual decline similar to the overall cohort, ending above 0.85 at year 8. The orange line represents Present psychotic features and shows a much steeper, rapid decline, dropping below 0.8 by year 4. Shaded areas around the lines indicate confidence intervals. A legend at the bottom right identifies the blue line as Absent and the orange line as Present, with a p-value less than 0.0001 noted below the legend.

Navigate back to Figure 2.

### Table 2 Long description

The table consists of eight columns: Predictor Category, Variable, Baseline H R (1 to 2 years), 95% C I, Time interaction H R, 95% C I, E-value (baseline), and P-value.

Time-invariant predictors:

* Age (per year): Baseline H R 1.009, 95% C I 1.007 to 1.011, E-value 1.10, P-value less than 0.001.

* Low income: Baseline H R 0.805, 95% C I 0.745 to 0.869, E-value 1.79, P-value less than 0.001.

* Foreign born: Baseline H R 1.049, 95% C I 0.905 to 1.217, E-value 1.28, P-value 0.524.

* Psychotic features: Baseline H R 3.183, 95% C I 2.929 to 3.458, E-value 5.82, P-value less than 0.001.

* Personality disorder: Baseline H R 1.206, 95% C I 1.118 to 1.301, E-value 1.70, P-value less than 0.001.

Time-varying predictors:

* Somatic comorbidities: Baseline H R 1.259, 95% C I 1.082 to 1.464, Time interaction H R 0.990, 95% C I 0.923 to 1.062, E-value 1.83, P-value 0.003.

* Substance use disorders: Baseline H R 0.947, 95% C I 0.796 to 1.127, Time interaction H R 1.180, 95% C I 1.077 to 1.294, E-value 1.30, P-value 0.539.

* Anxiety disorders: Baseline H R 0.407, 95% C I 0.344 to 0.481, Time interaction H R 1.306, 95% C I 1.196 to 1.427, E-value 4.35, P-value less than 0.001.

* Primary care visits: Baseline H R 0.985, 95% C I 0.983 to 0.988, Time interaction H R 1.008, 95% C I 1.007 to 1.008, E-value 1.14, P-value less than 0.001.

* Mental health visits: Baseline H R 1.013, 95% C I 1.009 to 1.018, Time interaction H R 1.002, 95% C I 1.000 to 1.004, E-value 1.13, P-value less than 0.001.

* M H hospitalizations: Baseline H R 1.141, 95% C I 1.118 to 1.165, Time interaction H R 0.937, 95% C I 0.924 to 0.950, E-value 1.54, P-value less than 0.001.

* Emergency visits: Baseline H R 1.004, 95% C I 0.995 to 1.014, Time interaction H R 1.000, 95% C I 0.995 to 1.005, E-value 1.07, P-value 0.376.

Navigate back to Table 2.

### Figure 3 Long description

The graph plots Hazard Ratio (H R) on a logarithmic Y-axis ranging from 0.3 to 3.0, against Time Since M D D Diagnosis on the X-axis with four intervals: 0-2 years, 2-5 years, 5-10 years, and >10 years. A dashed horizontal line at H R 1.0 indicates no effect.

* Substance Use Disorders (light blue) starts slightly below 1.0 and shows a steady linear increase, reaching approximately 1.5 at the >10 year mark.

* Anxiety Disorders (orange) starts as a protective factor at H R 0.4 and shows a steep upward trend, approaching H R 0.9 by the final period.

* Mental Health Hospitalizations (green) starts above 1.0 and shows a gradual decline, crossing below the 1.0 threshold after 5-10 years.

* Mental Health Outpatient Visits (dark blue) and Primary Care Visits (pink) both remain stable and clustered tightly around the H R 1.0 baseline throughout all time periods.

Each data point includes vertical error bars representing the 95 percent Confidence Interval (C I). The C I for Substance Use Disorders and Anxiety Disorders widens significantly in the later time periods.

Navigate back to Figure 3.

## References

[1] GBD. Global, regional, and national incidence, prevalence, and years lived with disability for 310 diseases and injuries, 1990–2015: a systematic analysis for the global burden of disease study 2015. Lancet. 2015;388(10053):1545–602. 10.1016/S0140-6736(16)31678-6. PMC5055577 27733282

[2] Ferrari AJ, Stockings E, Khoo JP, Erskine HE, Degenhardt L, Vos T, et al. The prevalence and burden of bipolar disorder: findings from the global burden of disease study 2013. Bipolar Disord. 2016;18(5):440–50. 10.1111/BDI.12423. 27566286

[3] Oliva V, Fico G, De Prisco M, Gonda X, Rosa AR, Vieta E. Bipolar disorders: an update on critical aspects. The Lancet Regional Health Europe. 2024;48:101135. 10.1016/J.LANEPE.2024.101135. 39811787 PMC11732062

[4] Nierenberg AA, Agustini B, Köhler-Forsberg O, Cusin C, Katz D, Sylvia LG, et al. Diagnosis and treatment of bipolar disorder. JAMA. 2023;330(14):1370–80. 10.1001/JAMA.2023.18588. 37815563

[5] Vieta E, Salagre E, Grande I, Carvalho AF, Fernandes BS, Berk M, et al. Early intervention in bipolar disorder. Am J Psychiatry. 2018;175(5):411–26. 10.1176/appi.ajp.2017.17090972. 29361850

[6] Dome P, Rihmer Z, Gonda X. Suicide risk in bipolar disorder: a brief review. Medicina (kaunas). 2019;55(8):403. 10.3390/medicina55080403. 31344941 PMC6723289

[7] McIntyre RS, Laliberté F, Germain G, MacKnight SD, Gillard P, Harrington A. The real-world health resource use and costs of misdiagnosing bipolar I disorder. J Affect Disord. 2022;316:26–33. 10.1016/j.jad.2022.07.069. 35952932

[8] Keramatian K, Pinto JV, Schaffer A, Sharma V, Beaulieu S, Parikh SV, et al. Clinical and demographic factors associated with delayed diagnosis of bipolar disorder: data from health outcomes and patient evaluations in bipolar disorder (HOPE-BD) study. J Affect Disord. 2022;296:506–13. 10.1016/J.JAD.2021.09.094. 34606817

[9] Lublóy Á, Keresztúri JL, Németh A, Mihalicza P. Exploring factors of diagnostic delay for patients with bipolar disorder: a population-based cohort study. BMC Psychiatry. 2020;20:1–17. 10.1186/S12888-020-2483-Y/TABLES/4. 32075625 PMC7031950

[10] Keramatian K, Pinto JV, Tsang VWL, Chakrabarty T, Yatham LN. Duration of untreated or undiagnosed bipolar disorder and clinical characteristics and outcomes: systematic review and meta-analysis. Br J Psychiatry. 2025;227(3):622–32. 10.1192/BJP.2025.63. 40337852

[11] Service SK, De La Hoz JF, Diaz-Zuluaga AM, Arias A, Pimplaskar A, Luu C, et al. Predicting diagnostic conversion from major depressive disorder to bipolar disorder: an EHR based study from Colombia. Bipolar Disord. 2025;27(1):47–56. 10.1111/BDI.13512. 39666719 PMC11848012

[12] Scott J, Graham A, Yung A, Morgan C, Bellivier F, Etain B. A systematic review and meta-analysis of delayed help-seeking, delayed diagnosis and duration of untreated illness in bipolar disorders. Acta Psychiatr Scand. 2022;146(5):389–405. 10.1111/ACPS.13490. 36018259

[13] Fico G, Vieta E. Antidepressant use in bipolar disorder: shifting focus from ‘whether’ to ‘whom.’. Eur Neuropsychopharmacol. 2024;84:1–2. 10.1016/j.euroneuro.2024.04.004. 38640608

[14] Nestsiarovich A, Reps JM, Matheny ME, DuVall SL, Lynch KE, Beaton M, et al. Predictors of diagnostic transition from major depressive disorder to bipolar disorder: a retrospective observational network study. Transl Psychiatry. 2021;11:642. 10.1038/s41398-021-01760-6. 34930903 PMC8688463

[15] Rhee SJ, Ohlsson H, Sundquist J, Sundquist K, Kendler KS. Predictors of diagnostic conversion from major depression to bipolar disorder: a Swedish national longitudinal study. Psychol Med. 2023;53(16):7805–16. 10.1017/S0033291723001848. 37427550 PMC10755232

[16] Musliner KL, Østergaard SD. Patterns and predictors of conversion to bipolar disorder in 91 587 individuals diagnosed with unipolar depression. Acta Psychiatr Scand. 2018;137(5):422–32. 10.1111/acps.12869. 29498031

[17] Hu YH, Chen K, Chang IC, Shen CC. Critical predictors for the early detection of conversion from unipolar major depressive disorder to bipolar disorder: Nationwide population-based retrospective cohort study. JMIR Med Inform. 2020;8((4)): e14278 10.2196/14278. 32242821 PMC7165312

[18] Kim H, Kim Y, Baek JH, Fava M, Mischoulon D, Nierenberg AA, et al. Predictive factors of diagnostic conversion from major depressive disorder to bipolar disorder in young adults ages 19–34: a nationwide population study in South Korea. J Affect Disord. 2020;265:52–8. 10.1016/j.jad.2020.01.009. 31957692

[19] de OJP, Jansen K, Cardoso T de A, Mondin TC, Souza LD de M, da SRA, et al. Predictors of conversion from major depressive disorder to bipolar disorder. Psychiatry Res. 2021;297: 10.1016/j.psychres.2021.113740. 33493732

[20] Xu Z, Chen L, Hu Y, Shen T, Chen Z, Tan T, et al. A predictive model of risk factors for conversion from major depressive disorder to bipolar disorder based on clinical characteristics and circadian rhythm gene polymorphisms. Front Psych. 2022;13:843400 10.3389/fpsyt.2022.843400. PMC9309512 35898634

[21] Tondo L, Visioli C, Preti A, Baldessarini RJ. Bipolar disorders following initial depression: Modeling predictive clinical factors. J Affect Disord. 2014;167:44–9. 10.1016/J.JAD.2014.05.043. 25082113

[22] Kessing LV, Willer I, Andersen PK, Bukh JD. Rate and predictors of conversion from unipolar to bipolar disorder: a systematic review and meta-analysis. Bipolar Disord. 2017;19(5):324–35. 10.1111/bdi.12513. 28714575

[23] Ratheesh A, Davey C, Hetrick S, Alvarez-Jimenez M, Voutier C, Bechdolf A, et al. A systematic review and meta-analysis of prospective transition from major depression to bipolar disorder. Acta Psychiatr Scand. 2017;135(4):273–84. 10.1111/acps.12686. 28097648

[24] Salazar de Pablo G, Perez-Rodriguez V, de Otazu Olivares J, Camacho-Rubio J, Sharma A, Catalán A, et al. Development and predictors of bipolar disorder in children and adolescents with depressive disorders: a systematic review, meta-analysis, and meta-regression. Eur Psychiatry. 2025;68(1): e16 10.1192/j.eurpsy.2024.1814. 39773691 PMC11822963

[25] Martini J, Bröckel KL, Leopold K, Berndt C, Sauer C, Maicher B, et al. Young people at risk for developing bipolar disorder: two-year findings from the multicenter prospective, naturalistic early-BipoLife study. Eur Neuropsychopharmacol. 2024;78:43–53. 10.1016/j.euroneuro.2023.10.001. 37913697

[26] De Prisco M, Oliva V, Fico G, Mas A, Valenzuela-Pascual C, Montejo L, et al. The PADRIS-PRESTO cohort: a comprehensive population-based study on mental health in Catalonia. Eur Psychiatry. 2025;68 10.1192/J.EURPSY.2025.10103. PMC12538175 40970440

[27] von Elm E, Altman DG, Egger M, Pocock SJ, Gøtzsche PC, Vandenbroucke JP, et al. The strengthening the reporting of observational studies in epidemiology (STROBE) statement: guidelines for reporting observational studies. Int J Surg. 2014;12(12):1495–9. 10.1016/j.ijsu.2014.07.013. 25046131

[28] Miola A, Ercis M, Pazdernik VK, Fuentes Salgado M, Ortiz-Orendain J, Gardea-Reséndez M, et al. Association between exposure to antidepressants and stimulants and age at onset of mania or psychosis: a retrospective population-based cohort study. Eur Neuropsychopharmacol. 2024;89:15–23. 10.1016/j.euroneuro.2024.07.015. 39226722

[29] Bartoli F, Cavaleri D, Bassetti C, Broccia M, Crocamo C, Malhi GS, et al. Adolescent cannabis use and onset of bipolar disorder: gaining causal clarity by viewing the evidence through the Bradford Hill lens. CNS Spectr. 2025;30((1)):e49 10.1017/S1092852925100345. 40534313 PMC13064723

[30] Bartoli F, Cavaleri D, Crocamo C. Artificial intelligence and bipolar disorder: applications of machine learning models for diagnosis, treatment, and outcome prediction. Alpha Psychiatry. 2025;26. 10.31083/ap44494. PMC12593754 41209509

[31] Jorgensen A, Sloth MMB, Larsen EN, Osler M, Kessing LV. Prescription sequences in bipolar disorder – a nationwide Danish register-based study of 19,927 individuals followed for 10 years. Eur Neuropsychopharmacol. 2025;93:51–7. 10.1016/j.euroneuro.2025.01.008. 39955809

[32] Akiskal HS, Benazzi F. Atypical depression: a variant of bipolar II or a bridge between unipolar and bipolar II? J Affect Disord. 2005;84(2-3):209–17. 10.1016/j.jad.2004.05.004. 15708418

[33] Berk M, Corrales A, Trisno R, Dodd S, Yatham LN, Vieta E, et al. Bipolar II disorder: a state-of-the-art review. World Psychiatry. 2025;24(2):175–89. 10.1002/wps.21300. 40371769 PMC12079553

[34] Salvetat N, Checa-Robles FJ, Delacrétaz A, Cayzac C, Dubuc B, Vetter D, et al. AI algorithm combined with RNA editing-based blood biomarkers to discriminate bipolar from major depressive disorders in an external validation multicentric cohort. J Affect Disord. 2024;356:385–93. 10.1016/j.jad.2024.04.022. 38615844

[35] Martinuzzi E, Barbosa S, Courtet P, Olié E, Guillaume S, Ibrahim EC, et al. Blood cytokines differentiate bipolar disorder and major depressive disorder during a major depressive episode: initial discovery and independent sample replication. Brain Behav Immun Health. 2021;13 10.1016/j.bbih.2021.100232. PMC8474674 34589747

[36] Zhu Y, Wu X, Liu H, Niu Z, Zhao J, Wang F, et al. Employing biochemical biomarkers for building decision tree models to predict bipolar disorder from major depressive disorder. J Affect Disord. 2022;308:190–8. 10.1016/j.jad.2022.03.080. 35439462

[37] Musliner KL, Krebs MD, Albiñana C, Vilhjalmsson B, Agerbo E, Zandi PP, et al. Polygenic risk and progression to bipolar or psychotic disorders among individuals diagnosed with unipolar depression in early life. Am J Psychiatry. 2020;177(10):936–43. 10.1176/APPI.AJP.2020.19111195. 32660297

[38] Biere S, Kranz TM, Matura S, Petrova K, Streit F, Chiocchetti AG, et al. Risk stratification for bipolar disorder using polygenic risk scores among Young high-risk adults. Front Psych. 2020;11 10.3389/FPSYT.2020.552532. PMC7653940 33192665

[39] De Almeida JRC, Phillips ML. Distinguishing between unipolar depression and bipolar depression: current and future clinical and neuroimaging perspectives. Biol Psychiatry. 2013;73(2):111–8. 10.1016/J.BIOPSYCH.2012.06.010. 22784485 PMC3494754

[40] Roberts G, Perry A, Lord A, Frankland A, Leung V, Holmes-Preston E, et al. Structural dysconnectivity of key cognitive and emotional hubs in young people at high genetic risk for bipolar disorder. Mol Psychiatry. 2018;23(2):413–21. 10.1038/MP.2016.216. 27994220 PMC5794888

