# Supplementary material for: Predictors of conversion from major depressive disorder to bipolar disorder: A population-based PADRIS-PRESTO cohort study in Catalonia
Source: Eur Psychiatry. 2026 Jul 2;69(1):e75. doi: 10.1192/j.eurpsy.2026.12235 (PMC13435152; doi:10.1192/j.eurpsy.2026.12235)
Supplement: Supplementary file 1 [file S0924933826122354sup001.zip › Supplementary_Tables(S1-S5).docx]

# Supplementary Table S1. Complete Time-Varying Cox Regression Model Coefficients

*Clinical Model for Bipolar Disorder Conversion from Major Depressive Disorder*

| **Variable** | **Coefficient** | **SE** | **95% CI** | **HR** | **P-value** |
| --- | --- | --- | --- | --- | --- |
| Age (per year) | 0.009 | 0.001 | (0.01–0.01) | 1.009 | <0.001 |
| Low income | -0.217 | 0.039 | (-0.29–-0.14) | 0.805 | <0.001 |
| Foreign born | 0.048 | 0.076 | (-0.10–0.20) | 1.049 | 0.524 |
| Somatic comorbidities | 0.230 | 0.077 | (0.08–0.38) | 1.259 | 0.003 |
| Time period (baseline) | — | 0.000 | — | — | — |
| Substance use disorder | -0.055 | 0.089 | (-0.23–0.12) | 0.947 | 0.539 |
| Anxiety disorder | -0.899 | 0.085 | (-1.07–-0.73) | 0.407 | <0.001 |
| Psychotic features | 1.158 | 0.042 | (1.07–1.24) | 3.183 | <0.001 |
| Personality disorder | 0.187 | 0.039 | (0.11–0.26) | 1.206 | <0.001 |
| Primary care visits (per visit) | -0.015 | 0.001 | (-0.02–-0.01) | 0.985 | <0.001 |
| Mental health visits (per visit) | 0.013 | 0.002 | (0.01–0.02) | 1.013 | <0.001 |
| MH hospitalizations (per admission) | 0.132 | 0.011 | (0.11–0.15) | 1.141 | <0.001 |
| Emergency visits (per visit) | 0.004 | 0.005 | (-0.01–0.01) | 1.004 | 0.376 |
| × Time period interaction: Somatic comorbidities | -0.010 | 0.036 | (-0.08–0.06) | 0.990 | 0.780 |
| × Time period interaction: Substance use disorder | 0.166 | 0.047 | (0.07–0.26) | 1.180 | <0.001 |
| × Time period interaction: Anxiety disorder | 0.267 | 0.045 | (0.18–0.36) | 1.306 | <0.001 |
| × Time period interaction: Primary care visits (per visit) | 0.008 | 0.000 | (0.01–0.01) | 1.008 | <0.001 |
| × Time period interaction: Mental health visits (per visit) | 0.002 | 0.001 | (0.00–0.00) | 1.002 | 0.035 |
| × Time period interaction: MH hospitalizations (per admission) | -0.065 | 0.007 | (-0.08–-0.05) | 0.937 | <0.001 |
| × Time period interaction: Emergency visits (per visit) | -0.000 | 0.002 | (-0.00–0.00) | 1.000 | 0.985 |

**Table S1.** Time period coded as continuous variable (1=1-2 years, 2=2-5 years, 3=5+ years). Interaction terms show how effects change per time period. Sex, urban residence, and suicide attempts were handled as stratification variables (no coefficients estimated). Model includes 52,001 patients with 3,958 conversion events. SE, standard error; CI, confidence interval; HR, hazard ratio; MH, mental health.

# Supplementary Table S2. Proportional Hazards Assumption Testing

*Schoenfeld Residual Test Results for Clinical Model*

| **Variable** | **χ²** | **df** | **P-value** | **PH Violation?** |
| --- | --- | --- | --- | --- |
| Age | 0.96 | 1 | 0.327 | No |
| Sex (male) | 0.65 | 1 | 0.421 | No |
| Low income | 0.46 | 1 | 0.499 | No |
| Urban residence | 4.18 | 1 | 0.041 | Yes |
| Foreign born | 0.00 | 1 | 0.963 | No |
| Somatic comorbidities | 28.80 | 1 | <0.001 | Yes |
| Substance use disorder | 18.03 | 1 | <0.001 | Yes |
| Anxiety disorder | 53.01 | 1 | <0.001 | Yes |
| Psychotic features | 3.40 | 1 | 0.065 | No |
| Personality disorder | 0.21 | 1 | 0.650 | No |
| Primary care visits | 353.81 | 1 | <0.001 | Yes |
| Mental health visits | 6.88 | 1 | 0.009 | Yes |
| Mental health hospitalizations | 38.31 | 1 | <0.001 | Yes |
| Emergency visits | 11.25 | 1 | 0.001 | Yes |
| Suicide attempts | 4.34 | 1 | 0.037 | Yes |
| **GLOBAL TEST** | **680.25** | **15** | **<0.001** | **Yes** |

χ², chi-square statistic; df, degrees of freedom; PH, proportional hazards.

**Table S2.** Schoenfeld residual tests assess whether predictor effects remain constant over time. Significant p-values (p<0.05) indicate violations of the proportional hazards assumption. Bonferroni-corrected α=0.003 was used for individual tests. Nine of 15 variables (60%) showed significant violations, necessitating time-varying coefficient modeling.

# Supplementary Table S3. E-values for Robustness to Unmeasured Confounding

*Time-Varying Cox Model Period 1 (1-2 years post-diagnosis)*

| **Variable** | **HR** | **95% CI** | **E-value** | **E-value (CI)** | **Interpretation** |
| --- | --- | --- | --- | --- | --- |
| Age (per year) | 1.009 | (1.01–1.01) | 1.11 | 1.09 | Minimal confounding needed |
| Low income | 0.805 | (0.74–0.87) | 1.90 | 1.64 | Moderate robustness |
| Psychotic features | 3.180 | (2.92–3.45) | 5.82 | 5.33 | Exceptional robustness |
| Personality disorder | 1.026 | (1.12–1.30) | 1.70 | 1.50 | Weak association |
| Substance use disorder | 0.950 | (0.80–1.13) | 1.27 | — | Non-significant baseline |
| Anxiety disorder | 0.407 | (0.34–0.48) | 4.35 | 2.97 | Very robust protective effect |
| Somatic comorbidities (per condition) | 1.280 | (1.17–1.40) | 2.35 | 2.07 | Moderate robustness |
| Primary care visits (per visit) | 1.002 | (1.00–1.00) | 1.03 | 1.02 | Minimal effect |
| Mental health visits (per visit) | 1.020 | (1.02–1.03) | 1.18 | 1.17 | Moderate robustness |
| MH hospitalizations (per admission) | 1.140 | (1.12–1.17) | 1.63 | 1.57 | Moderate robustness |
| Emergency visits (per visit) | 1.000 | (0.99–1.01) | 1.05 | — | Non-significant |

**Table S3.** E-values quantify robustness to unmeasured confounding. The E-value represents the minimum strength of association (on the risk ratio scale) that an unmeasured confounder would need to have with both the exposure and outcome to fully explain away the observed association, conditional on measured covariates. HR, hazard ratio; CI, confidence interval; MH, mental health.

# Supplementary Table S4. Comprehensive Model Validation Metrics

*Internal and Temporal Validation of Clinical Prediction Models*

| **Validation Type** | **Model** | **Metric** | **Value** | **Notes** |
| --- | --- | --- | --- | --- |
| Bootstrap Validation | Standard Cox | Original C-index | 0.669 | Before correction |
| Bootstrap Validation | Standard Cox | Optimism | 0.051 | 200 resamples |
| Bootstrap Validation | Standard Cox | Corrected C-index | 0.720 | 95% CI: 0.706-0.733 |
| Bootstrap Validation | Time-Varying Cox | Apparent C-index | 0.745 | Before correction |
| Bootstrap Validation | Time-Varying Cox | Optimism | 0.025 | 200 resamples |
| Bootstrap Validation | Time-Varying Cox | Corrected C-index | 0.720 | 95% CI: 0.706-0.733 |
| Temporal Validation | Time-Varying Cox | Training C (2010-2015) | 0.747 | n=28,875; 3,509 events |
| Temporal Validation | Time-Varying Cox | Validation C (2016-2019) | 0.741 | n=23,126; 449 events |
| Temporal Validation | Time-Varying Cox | Performance decline | 0.8% | Excellent stability |
| Time-Dependent AUC | Standard Cox | 2-year AUC | 0.623 | Early prediction |
| Time-Dependent AUC | Standard Cox | 5-year AUC | 0.657 | Mid-term prediction |
| Time-Dependent AUC | Standard Cox | 10-year AUC | 0.617 | Long-term prediction |
| Time-Dependent AUC | Time-Varying Cox | 2-year AUC | 0.503 | Patient-level aggregation |
| Time-Dependent AUC | Time-Varying Cox | 5-year AUC | 0.557 | Patient-level aggregation |
| Time-Dependent AUC | Time-Varying Cox | 10-year AUC | 0.575 | Patient-level aggregation |
| Calibration | Time-Varying Cox | Hosmer-Lemeshow χ² | 13.41 | df=8 |
| Calibration | Time-Varying Cox | P-value | 0.127 | No significant miscalibration |

**Table S4,** Based on 200 bootstrap resamples. Both standard and time-varying Cox models converge to identical optimism-corrected C-index (0.720), with time-varying model showing lower optimism (0.025 vs 0.051), indicating better model specification without overfitting. Model developed on 2010-2015 cohort and validated on 2016-2019 cohort without refitting. Minimal performance degradation (0.8%) demonstrates excellent generalizability across time periods despite evolving diagnostic practices. Standard Cox model shows higher time-point AUC values (0.623/0.657/0.617) compared to time-varying model (0.503/0.557/0.575) because time-varying predictions must be aggregated to patient-level, losing temporal information. The appropriate discrimination metric for time-varying models is the C-index on the time-split structure (0.745). See Supplementary Results Section A.6 for detailed explanation. Hosmer-Lemeshow test shows no significant miscalibration (p=0.127). Detailed calibration plots at 2, 5, and 10 years provided in Supplementary Figure S2. C, concordance index; CI, confidence interval; AUC, area under the curve; χ², chi-square statistic; df, degrees of freedom.

# Supplementary Table S5. Comparison of Standard vs Time-Varying Cox Models

*Clinical and General Model Performance Metrics*

| **Model** | **C-index** | **PH Violations (n)** | **Total Variables** |
| --- | --- | --- | --- |
| Clinical Model (Standard Cox) | 0.669 | 7/12 (58%) | 12 |
| **Clinical Model (Time-Varying Cox)** | **0.745** | **10/19 (53%)** | **19** |
| General Model (Standard Cox) | 0.874 | 13/17 (76%) | 17 |
| General Model (Time-Varying Cox) | 0.857 | 12/30 (40%) | 30 |

**Model specifications:** Clinical models include only pre-diagnosis patient characteristics and healthcare utilization. General models additionally include medication variables (antidepressants, antipsychotics, mood stabilizers, benzodiazepines). Clinical Time-Varying Cox model (highlighted) is presented as the primary analysis in the main manuscript. This model achieves excellent discrimination (C=0.745) without medication circularity bias and provides actionable predictions at initial MDD presentation. General models show higher apparent discrimination (C=0.874/0.857) but include medications that likely reflect clinician diagnostic suspicion (mood stabilizers, antipsychotics) rather than independent risk factors. These models are presented as sensitivity analyses in Supplementary Results Section B. Time-varying models show more PH violations than standard models because interactions with time period are themselves tested for proportional hazards. This is expected and does not indicate model misspecification. Complete PH testing results in Supplementary Table S2. C-index, concordance index; PH, proportional hazards.
