# Supplementary material for: Predictors of conversion from major depressive disorder to bipolar disorder: A population-based PADRIS-PRESTO cohort study in Catalonia
Source: Eur Psychiatry. 2026 Jul 2;69(1):e75. doi: 10.1192/j.eurpsy.2026.12235 (PMC13435152; doi:10.1192/j.eurpsy.2026.12235)
Supplement: Supplementary file 1 [file S0924933826122354sup001.zip › Supplementary_Figures(S1-S3).docx]

Supplementary Figure S1. Time-Varying Hazard Ratios Across Follow-Up Periods for Clinical and General Prediction Models

Figure S1.Time-varying hazard ratios (HRs) demonstrating temporal evolution of predictor effects across four follow-up periods (1–2 years, 2–5 years, and ≥5 years post‑MDD diagnosis). Panel A shows clinical model predictors that violated proportional hazards assumptions and required time-varying coefficients. Panel B shows medication variables from the general model demonstrating circularity bias, with prescriptions likely reflecting clinician diagnostic suspicion rather than independent risk factors. HR, hazard ratio; MDD, major depressive disorder; mh, mental health; y, years; pre, pre-diagnosis baseline period.

# Supplementary Figure S2: Calibration plots at 2, 5, and 10 years - shows calibration of predicted vs observed risks

Figure S2. Calibration assessment comparing predicted versus observed bipolar disorder conversion risk at three clinically relevant time points. Each plot shows the relationship between model-predicted risk (x-axis) and observed conversion frequency (y-axis) using locally weighted regression (LOESS). Perfect calibration (diagonal dashed line) indicates exact agreement between predictions and observations. Calibration slopes and intercepts quantify systematic over- or under-prediction patterns across the risk spectrum.

# Supplementary Figure S3. Machine Learning Feature Importance Analysis from XGBoost Models

Figure S3. Feature importance rankings from gradient-boosted machine learning models (XGBoost) showing convergence with time-varying Cox regression results. Panel A displays the top 15 features from the clinical model (C-index=0.732), demonstrating that clinically accessible variables achieve strong discrimination without medication data. Panel B shows the top 15 features from the general model including medications (C-index=0.813), highlighting that medication variables dominate when included but likely reflect circularity bias. mh, mental health; pre, pre-diagnosis baseline period; n, number of.
