# Supplementary material for: Predictors of conversion from major depressive disorder to bipolar disorder: A population-based PADRIS-PRESTO cohort study in Catalonia
Source: Eur Psychiatry. 2026 Jul 2;69(1):e75. doi: 10.1192/j.eurpsy.2026.12235 (PMC13435152; doi:10.1192/j.eurpsy.2026.12235)
Supplement: Supplementary file 1 [file S0924933826122354sup001.zip › Supplementary_Methods.docx]

Supplementary Methods

Detailed Variable Definitions and Coding

Sociodemographic Variables

Age at MDD diagnosis was estimated from the five-year age range provided by PADRIS for anonymization purposes, using the midpoint of each age bracket as the point estimate. Low income status was defined using the PADRIS socioeconomic classification based on annual income categories. Patients were classified as low income if they fell into the <€18,000/year bracket or were classified as ‘Exempted’ from income reporting (a category that reflects economic vulnerability and exemption from healthcare copayments). Urban residence was derived from the healthcare territorial unit (Sector Sanitari) provided by PADRIS, mapped to the Spanish National Statistics Institute (INE) municipal classification. Municipalities exceeding 10,000 inhabitants were classified as urban. This variable was not directly provided by PADRIS and was constructed based on healthcare sector geocoding. Foreign-born status was determined from nationality recorded in the healthcare registry provided by PADRIS, with patients from outside Spain classified as foreign-born.

Clinical Variables

Medical comorbidities were determined based on the physical diagnoses participants received during during the observation period. Psychotic features were identified by the presence of ICD-10 codes F32.3 or F33.3 (severe depressive episode with psychotic symptoms) during any depressive episode. Anxiety disorders included generalized anxiety disorder (F41.1), panic disorder (F41.0), agoraphobia (F40.0), social phobia (F40.1), specific phobias (F40.2), and unspecified anxiety disorder (F41.9). Substance use disorders encompassed alcohol use disorders (F10.x) and drug use disorders (F11.x-F19.x). Personality disorders included all F60.x codes. Somatic comorbidities were operationalized as a count variable (range 0–6) of the following conditions present during the observation period: diabetes mellitus, hypertension (essential or secondary), chronic obstructive pulmonary disease (COPD), asthma, heart failure, congestive heart failure, and chronic kidney disease.

Suicide attempts were identified from emergency department records coded as intentional self-harm (ICD-10 X60-X84) or psychiatric hospitalization records documenting suicide attempts. Due to the very low prevalence of recorded suicide attempts in the cohort and resulting model convergence, this variable was excluded from multivariable models.

Healthcare Utilization Variables

Primary care visits were counted from the primary care registry during the one-year pre-index period, including both in-person and telephone consultations. Mental health visits included outpatient psychiatry and psychology appointments recorded in specialized mental health services. Emergency department visits were counted from emergency service records with any mental health-related chief complaint or diagnosis. Psychiatric hospitalizations included all inpatient admissions to psychiatric units or general hospital psychiatric wards, counted as separate episodes even if occurring during the same hospital stay.

Medication Variables (General Model Only)

Psychotropic medication use was ascertained from pharmacy dispensing records during the one-year pre-index period. Medications were classified by Anatomical Therapeutic Chemical (ATC) codes: antidepressants (N06A), antipsychotics (N05A excluding lithium), mood stabilizers (N03AG01 valproate, N03AF01 carbamazepine, N05AN01 lithium), and benzodiazepines (N05BA, N05CD). The number of psychotropic medication classes variable counted the total number of distinct therapeutic classes received by each patient (range: 0-4). Medication variables were included only in the general model due to concerns about confounding by indication, as medication prescription is directly influenced by symptom severity.

Missing Data Assessment and Handling

Missing data were minimal (<2%) for all predictor variables except foreign-born status (5.8% missing). Complete case analysis was performed as the primary approach given the low missingness rate. Sensitivity analyses using multiple imputation by chained equations (20 imputed datasets) yielded virtually identical results (all hazard ratios within 3% of complete case estimates), supporting the robustness of our findings to missing data assumptions.

Descriptive baseline comparisons

For descriptive comparisons (Table 1), continuous variables were summarized as means with standard deviations and 95% confidence intervals; categorical variables as frequencies with percentages and 95% confidence intervals calculated using the Wilson score method. Effect sizes were quantified using standardized differences to facilitate comparison of imbalance across variables with different scales. For continuous variables, Cohen's d was calculated as the absolute mean difference divided by the pooled standard deviation. For binary and categorical variables, Austin's standardized difference was used, calculated as d = |p₁ - p₂| / √[(p₁(1-p₁) + p₂(1-p₂))/2], where p₁ and p₂ represent proportions in each group (Austin, 2009). Values exceeding 0.10 indicate meaningful imbalance between groups. Group differences were tested using independent t-tests for continuous variables, chi-square tests for categorical variables, and Wilcoxon rank-sum tests for medians.

Proportional Hazards Assessment and Model Selection

Schoenfeld Residual Testing Framework

We conducted comprehensive proportional hazards testing using scaled Schoenfeld residuals with Kaplan-Meier transformation of time. Individual variable tests employed Bonferroni correction (α=0.05/k where k=number of predictors) to account for multiple testing. The global test statistic followed a chi-square distribution with degrees of freedom equal to the number of predictors.

Time-Varying Coefficient Implementation

For variables violating proportional hazards assumptions, we implemented time-varying coefficients by splitting follow-up into clinically meaningful periods (1-2, 2-5, and 5+ years) based on observed hazard function patterns and clinical considerations about diagnostic stability over time. The lower bound of period 1 reflects the ≥1 year follow-up inclusion criterion, while maximum observable follow-up was approximately 9 years given the 2010-2019 study period. The survSplit() function created an extended dataset with time-period indicators, allowing interaction terms between predictors and time periods. Model selection between constant and time-varying specifications used likelihood ratio tests with Akaike Information Criterion (AIC) for non-nested comparisons.

Stratification Approach for Non-Proportional Baseline Hazards

Variables with substantial between-group heterogeneity in baseline hazards (sex, urban residence, suicide attempts) were addressed through stratified Cox regression. This approach creates separate baseline hazard functions for each stratum combination while maintaining common regression coefficients across strata, thereby preserving statistical power while accounting for baseline heterogeneity. Stratification is appropriate when the focus is on covariate effects rather than the baseline hazard itself.

Model Performance Assessment and Validation

Discrimination Metrics

**Concordance Index (C-index).** The C-index quantifies the probability that, for a randomly selected pair of patients where one experiences the outcome, the model assigns a higher risk to that patient. For time-varying Cox models, the C-index was calculated on the time-split data structure, which appropriately accounts for changing covariate effects over time. Values range from 0.5 (random prediction) to 1.0 (perfect discrimination).

**Time-Dependent Area Under the Curve (AUC).** Time-dependent AUC evaluates discriminative ability at specific time points (2, 5, and 10 years). For time-varying Cox models, we aggregated predictions to patient level by using the most recent (final time period) linear predictor for each individual. We calculated time-dependent AUC using the Kaplan-Meier method implemented in the survival ROC package. Importantly, patient-level AUC values from time-varying models may appear lower than standard Cox models because aggregation to a single prediction per patient necessarily loses temporal granularity. The appropriate discrimination metric for time-varying Cox models is the C-index calculated on the time-varying structure, as this properly accounts for temporally changing effects.

Bootstrap Internal Validation

We performed bootstrap internal validation with 200 resamples to assess model optimism and estimate the optimism-corrected C-index. Bootstrap resamples were drawn with replacement from the original dataset, maintaining the same sample size. For each bootstrap sample, we fit the time-varying Cox model and calculated the apparent C-index. The optimism was estimated as the difference between the apparent C-index on the original data and the mean bootstrap C-index. The optimism-corrected C-index provides an estimate of expected model performance on new data from the same population. Low optimism values (typically <0.05) indicate good model stability and suggest the model will generalize well.

Calibration Assessment

For time-varying Cox models, traditional calibration metrics based on predicted probabilities are challenging to interpret because covariate effects change over time, making single-number probability predictions conceptually problematic. Instead, we assessed calibration by examining the relationship between model-predicted risk (linear predictor) and observed event rates across risk deciles. We calculated the Spearman correlation between risk group rank and observed event rate to quantify the strength of the risk-outcome relationship. A strong positive correlation (ρ > 0.90) indicates that the model appropriately discriminates between low and high-risk patients, with monotonically increasing event rates across risk groups.

Temporal Validation

We attempted temporal validation by splitting the cohort based on MDD diagnosis year, with training on 2010-2015 data (n=28,875) and validation on 2016-2019 data (n=23,126). However, the stratified nature of our Cox model combined with temporal differences in stratification variable combinations prevented direct validation. Specifically, some stratification combinations present in the validation cohort were not observed in the training period, causing model fitting issues. This is a known limitation of stratified Cox models when applied to temporally separated cohorts and does not reflect model instability. As an alternative, we relied on bootstrap internal validation optimism estimates to assess model stability and generalizability.

Sensitivity and Robustness Analyses

Competing Risks Framework

Death as a competing event was addressed using Fine-Gray subdistribution hazard models. We compared cause-specific hazard ratios (standard Cox regression) with subdistribution hazard ratios to assess whether differential mortality patterns biased our estimates. The cumulative incidence function was calculated accounting for the competing risk of death using the Aalen-Johansen estimator.

E-value Calculation Methods

E-values quantify the minimum strength of association that an unmeasured confounder would require with both the exposure and outcome to explain away observed associations. We calculated E-values using the formula: E = HR + sqrt(HR × (HR - 1)) for the point estimate, with confidence interval bounds calculated similarly. E-values were computed conditional on measured covariates using the EValue R package with standardized mortality ratio approximation.

Machine Learning Implementation

Algorithm Specifications

Machine learning models employed identical predictor sets and temporal data splits as the primary Cox regression for direct comparison. LASSO logistic regression used 10-fold cross-validation for lambda selection with alpha=1. Random Forest employed 500 trees with mtry=sqrt(p) and minimum node size of 50 for stability. XGBoost parameters included max_depth=6, eta=0.3, nrounds selected via early stopping with 20-round patience, and gamma=0 for no regularization.

Feature Importance Extraction

Variable importance metrics were standardized across algorithms for comparison. LASSO importance used absolute coefficient values after standardization. Random Forest employed mean decrease in node impurity (Gini index). XGBoost importance represented gain (improvement in accuracy) when using each feature for splits. All importance scores were normalized to 0-100 scale with the most important feature set to 100.

Statistical Software and Package Versions

All analyses were conducted in R version 4.5.1 (2024-06-14). Key packages: survival version 3.5-7 (Cox models, survSplit, time-varying coefficients); survminer version 0.4.9 (Kaplan-Meier plots, log-rank tests); rms version 6.7-1 (model validation, calibration); boot version 1.3-28.1 (bootstrap resampling); survivalROC version 1.0.3.1 (time-dependent AUC); cmprsk version 2.2-11 (Fine-Gray competing risks models); EValue version 4.1.3 (sensitivity to unmeasured confounding); glmnet version 4.1-8 (LASSO regression); randomForest version 4.7-1.1 (Random Forest); xgboost version 1.7.5.1 (gradient boosting); pROC version 1.18.4 (ROC curve analysis, AUC calculation); data.table version 1.14.8 (data manipulation); ggplot2 version 3.4.4 (visualization).
