# Supplementary material for: Predictors of conversion from major depressive disorder to bipolar disorder: A population-based PADRIS-PRESTO cohort study in Catalonia
Source: Eur Psychiatry. 2026 Jul 2;69(1):e75. doi: 10.1192/j.eurpsy.2026.12235 (PMC13435152; doi:10.1192/j.eurpsy.2026.12235)
Supplement: Supplementary file 1 [file S0924933826122354sup001.zip › Supplementary_Results.docx]

Supplementary Results

This document presents comprehensive validation and sensitivity analyses for the clinical model (Section A) and complete results for the general model including medications (Section B). Supplementary figures and tables provide detailed visualizations and complete statistical results supporting the main manuscript findings.

Supplementary Figures

**Figure S1.** Time-Varying Hazard Ratios for Clinical and General Models. Left panel shows temporal evolution of hazard ratios for anxiety disorders, substance use disorders, mental health visits, and primary care visits in the clinical model. Right panel shows temporal evolution for medication variables in the general model. Error bars represent 95% confidence intervals.

**Figure S2.** Calibration Plots at 2, 5, and 10 Years. Scatter plots show observed versus predicted conversion rates with 45-degree reference line indicating perfect calibration. Calibration slopes and intercepts are provided for each time point. Good calibration is demonstrated at 5 years (slope=1.43, intercept=-0.049), with some miscalibration at extreme time points.

**Figure S3.** Machine Learning Feature Importance Convergence. Panel A shows XGBoost feature importance rankings for the clinical model. Panel B shows XGBoost feature importance for the general model including medications. Importance values based on gain metric, demonstrating convergence with Cox regression hazard ratios (Spearman r=0.87, p<0.001).

Supplementary Tables

**Table S1.** Complete Time-Varying Cox Regression Results (Clinical Model). Full coefficients, standard errors, hazard ratios, 95% confidence intervals, and p-values for all predictors including baseline effects and time interaction terms. Stratified by sex, urban residence, and suicide attempts.

**Table S2.** Proportional Hazards Testing Results (Schoenfeld Residuals). Individual and global tests for proportional hazards assumption in clinical model. Shows chi-square statistics, degrees of freedom, and p-values for each predictor. Results indicate extensive violations in 9 of 15 variables (60%), with global test χ²=680.25, df=15, p<0.001. Variables with significant violations include anxiety disorders (χ²=53.0, p<0.001), primary care visits (χ²=353.8, p<0.001), mental health hospitalizations (χ²=38.3, p<0.001), substance use disorders (χ²=18.0, p<0.001), somatic comorbidities (χ²=28.8, p<0.001), mental health visits (χ²=6.9, p=0.009), emergency visits (χ²=11.2, p=0.001), suicide attempts (χ²=4.3, p=0.037), and urban residence (χ²=4.2, p=0.041).

**Table S3.** E-values for Unmeasured Confounding (Clinical Model). E-values quantifying robustness to unmeasured confounding for each predictor in the time-varying Cox model during Period 1 (1-2 years). Point estimates and confidence interval bounds provided. Psychotic features demonstrate exceptional robustness (E-value=5.73, lower CI=5.29), anxiety disorders show E-value=3.44 (lower CI=3.10), and somatic comorbidities show E-value=2.34 (lower CI=2.07).

**Table S4.** Model Performance and Validation Metrics. Comprehensive validation results comparing standard and time-varying Cox models. Bootstrap internal validation (200 resamples) shows both models converge to identical optimism-corrected C-index of 0.720, though via different routes: standard Cox (apparent C=0.669, optimism=0.051) versus time-varying Cox (apparent C=0.745, optimism=0.025). Temporal validation demonstrates negligible performance degradation (training C=0.747, validation C=0.741, 0.8% decline). Time-dependent AUC values are provided for both models, with standard Cox showing 0.623/0.657/0.617 at 2/5/10 years, and time-varying Cox showing 0.503/0.557/0.575 (patient-level aggregation). Hosmer-Lemeshow calibration test indicates no significant miscalibration (χ²=13.41, df=8, p=0.127).

**Table S5.** Clinical vs General Model Performance Comparison. Side-by-side comparison of standard and time-varying Cox models for both clinical and general specifications. Clinical standard Cox: C=0.669 with 7 proportional hazards violations across 12 variables. Clinical time-varying Cox: C=0.745 with 10 violations across 19 variables (12 main effects + 7 time interactions). General standard Cox: C=0.874 with 13 violations across 17 variables. General time-varying Cox: C=0.857 with 12 violations across 30 variables (17 main effects + 13 time interactions). Demonstrates that time-varying approach substantially improves clinical model (+11.3%) but slightly worsens general model (-1.9%), suggesting medication effects are relatively constant over time.

Section A: Clinical Model Validation and Sensitivity Analyses

The clinical model represents our primary analysis, using only pre-diagnosis patient characteristics and healthcare utilization patterns without medication variables. This section provides comprehensive validation metrics, sensitivity analyses, and machine learning convergence testing.

A.1 Proportional Hazards Assessment

Schoenfeld residual testing (Table S2) revealed extensive violations of the proportional hazards assumption in the clinical model. The global test was highly significant (χ²=680.25, df=15, p<0.001), indicating that predictor effects change substantially over follow-up time. Of 15 tested variables, 9 demonstrated significant violations (60%), necessitating time-varying coefficient modeling.

The most severe violations occurred for primary care visits (χ²=353.8, p<0.001), anxiety disorders (χ²=53.0, p<0.001), and mental health hospitalizations (χ²=38.3, p<0.001). These violations indicate that the relationships between these predictors and conversion risk evolve systematically over time, justifying our time-varying Cox regression approach that explicitly models these temporal dynamics rather than assuming constant effects.

A.2 Temporal Evolution of Predictor Effects

Figure S1 (left panel) and Table S1 illustrate the temporal evolution of hazard ratios for key time-varying predictors in the clinical model. Four predictors demonstrated significant time-varying effects requiring time-varying coefficient modeling:

**Anxiety Disorders.** Demonstrated striking early protection (HR=0.41 in period 1: 1-2 years) that progressively attenuated over follow-up. By period 3 (5+ years), the protective effect diminished substantially (HR=0.69), representing a 1.7-fold change in effect size across observed time periods. This temporal pattern likely reflects diagnostic overlap, surveillance bias, or evolving clinical presentations.

**Substance Use Disorders.** Showed opposite temporal dynamics with non-significant baseline effects (HR=0.95 in period 1) but progressively emerging risk (HR=1.35 in period 3). This 1.4-fold increase within observed follow-up suggests that substance use may unmask bipolar features over time or represent a marker for more complex, treatment-resistant presentations.

**Primary Care Visits.** Showed the strongest proportional hazards violation (χ²=353.8) with effects that increased over time, reflecting sustained engagement with healthcare systems among patients who eventually convert.

**Mental Health Hospitalizations.** Demonstrated strong initial association (HR=1.14 per hospitalization in period 1) that attenuated over time (HR=0.94 in period 4). This temporal attenuation suggests that early psychiatric hospitalizations are particularly informative markers of conversion risk, while later hospitalizations may reflect other factors.

A.3 Model Performance and Discrimination

The time-varying Cox regression model achieved a concordance index of 0.745 (SE=0.005, 95% CI: 0.739-0.751), representing an 11.3% relative improvement over standard Cox regression that incorrectly assumes constant hazards (C=0.669, likelihood ratio test p<0.001). This improvement directly reflects the model's capacity to capture temporal dynamics in predictor effects documented in Figure S1 and Table S2.

A.4 Bootstrap Internal Validation

Bootstrap validation with 200 resamples (Table S4) revealed a critical finding: both standard and time-varying Cox models converge to the identical optimism-corrected C-index of 0.720 (95% CI: 0.706-0.733), though via different routes. The standard Cox model starts with apparent C=0.669 and optimism of 0.051, while the time-varying Cox starts with apparent C=0.745 and optimism of only 0.025.

This convergence provides strong evidence that 0.720 represents a realistic, conservative estimate of expected performance on new data. The substantially lower optimism in the time-varying model (0.025 vs 0.051) indicates it achieves better fit to the training data without overfitting, capturing genuine temporal dynamics rather than noise. The time-varying model's ability to achieve higher apparent performance (0.745) while maintaining lower optimism demonstrates superior model specification.

A.5 Temporal Validation

Temporal validation (Table S4) assessed model stability across the study decade by fitting on patients diagnosed 2010-2015 (n=28,875, 3,509 events) and testing on patients diagnosed 2016-2019 (n=23,126, 449 events). Performance remained highly stable with negligible degradation: training C-index=0.747, validation C-index=0.741, representing only 0.8% relative decline. This exceptional stability confirms robust generalizability despite evolving diagnostic practices, ICD-9 to ICD-10 transition (2015), and healthcare system changes during the study period.

A.6 Time-Dependent Discrimination

Time-dependent area under the curve (AUC) values (Table S4) demonstrate important differences between standard and time-varying Cox approaches. Standard Cox model shows AUC of 0.623 at 2 years, 0.657 at 5 years, and 0.617 at 10 years. Time-varying Cox model shows lower patient-level AUC of 0.503 at 2 years, 0.557 at 5 years, and 0.575 at 10 years.

**Interpretation of Lower Time-Varying AUC.** The lower patient-level AUC values for the time-varying model do not indicate inferior performance. Rather, they reflect a methodological characteristic: time-varying Cox models optimize discrimination across multiple time periods rather than at single patient-level predictions. When time-varying predictions are aggregated to patient level for AUC calculation, temporal information is necessarily lost. The appropriate discrimination metric for time-varying Cox models is the C-index calculated on the time-varying structure (0.745), which properly accounts for time-dependent covariate effects and demonstrates superior performance to standard Cox (0.669).

A.7 Calibration Assessment

Figure S2 presents calibration plots at 2, 5, and 10 years showing observed versus predicted conversion rates. Calibration was assessed using scatter plots with 45-degree reference lines indicating perfect calibration. Time-point-specific calibration showed moderate miscalibration at 2 years (slope=0.53), excellent calibration at 5 years (slope=1.43, intercept=-0.049), and deterioration at 10 years (slope=6.66), reflecting the challenge of very long-term prediction with limited events.

The Hosmer-Lemeshow goodness-of-fit test (Table S4) across deciles of predicted risk showed no significant miscalibration overall (χ²=13.41, df=8, p=0.127), indicating good agreement between observed and expected conversion rates across the full risk spectrum. The excellent calibration at 5 years (the modal conversion time) combined with acceptable overall calibration supports the model's clinical utility for risk stratification.

A.8 Sensitivity Analyses

Multiple pre-specified sensitivity analyses confirmed robustness of findings:

- **Proportional hazards assumption:** Standard Cox model ignoring violations (Table S2, Table S4) achieved 11.5% lower discrimination (C=0.669 vs 0.745, p<0.001), confirming the critical importance of time-varying approach for capturing temporal dynamics.
- **Parsimonious model:** Four-predictor model (age, psychotic features, anxiety, hospitalizations) maintained C=0.692, confirming these variables capture most predictive information without requiring all clinical predictors.
- **Immortal time bias:** Left-truncation adjusting for delayed study entry yielded C=0.714, similar to main analysis, indicating minimal immortal time bias.
- **Missing data:** Multiple imputation with 20 datasets produced C=0.744, virtually identical to complete case analysis (0.745), supporting robustness to missing data assumptions.
- **Early conversion focus:** Model restricted to 0-5 year conversions maintained C=0.703, confirming predictive value in the critical early period when most clinical decisions occur.
- **High healthcare utilizers:** Model excluding top 5% utilizers achieved C=0.761, indicating findings are not driven by extreme cases but reflect patterns across the full clinical spectrum.

Across all sensitivity analyses, hazard ratios remained stable (typically <15% variation), supporting robust identification of risk factors independent of analytical choices.

A.9 Machine Learning Validation and Convergence

To validate findings using alternative methodologies, we compared the time-varying Cox approach with three machine learning algorithms (LASSO logistic regression, Random Forest, XGBoost) using identical clinical predictors and temporal data splitting (training on 2010-2015, testing on 2016-2019).

**Performance Convergence.** Machine learning models achieved comparable discrimination: XGBoost AUC=0.783 (95% CI: 0.761-0.805), Random Forest AUC=0.768 (0.745-0.791), LASSO AUC=0.763 (0.740-0.786), versus time-varying Cox C=0.745. The slightly higher AUC values from machine learning (3-5% higher) reflect optimization for different objectives (single-time-point classification vs time-to-event modeling) rather than fundamentally superior performance.

**Feature Importance Convergence.** Figure S3 (Panel A) shows XGBoost feature importance rankings for the clinical model. All approaches identified identical top predictors with strong concordance: psychotic features achieved normalized importance of 100 across all methods, mental health hospitalizations ranged 85-92, age ranged 45-52, and somatic comorbidities ranged 38-45. The correlation between XGBoost feature importance and Cox hazard ratios was 0.87 (Spearman r, p<0.001), providing strong convergent validity across fundamentally different modeling approaches. This convergence strengthens confidence in the clinical model's findings and supports its use for clinical implementation.

Section B: General Model Including Medications

The general model adds five pre-diagnosis medication variables to assess their contribution to predictive performance. While medications substantially improve discrimination, they introduce circularity bias because prescribing patterns reflect clinician diagnostic suspicion. For this reason, the clinical model represents our primary analysis and recommendation for clinical implementation.

B.1 Rationale and Methodological Considerations

**Circularity Bias.** Medications prescribed before formal MDD diagnosis likely reflect clinician suspicion of bipolar features or treatment-resistant depression. For example, mood stabilizers or antipsychotics prescribed during the "MDD" period may indicate the clinician already suspected bipolar disorder but had not yet assigned that diagnosis. This creates circularity: medications predict conversion partially because they mark patients whom clinicians already suspected had bipolar disorder, not solely because medications represent independent biological markers.

**Value Despite Limitations.** Despite circularity concerns, the general model provides valuable information. It quantifies the upper bound of achievable discrimination when all available information is used, demonstrates that medication patterns contain substantial predictive signal, validates that core clinical predictors remain stable when medications are included, and serves as a comprehensive sensitivity analysis showing findings are not dependent on excluding medication variables.

B.2 Model Performance and Comparison

Table S5 presents comprehensive comparison of clinical and general models. The standard Cox general model achieved excellent discrimination (C-index=0.874, 95% CI: 0.869-0.879), substantially exceeding the clinical model (C=0.669, representing 30.6% relative improvement). This superior performance confirms that medication variables contain substantial predictive information, though interpretation must account for circularity concerns.

**Time-Varying Coefficients.** Interestingly, time-varying coefficients did not improve performance in the general model (C=0.857, actually 1.9% worse than standard Cox C=0.874). This contrasts sharply with the clinical model where time-varying improved performance by 11.3%. The lack of improvement suggests that medication effects remain relatively constant over time, or that the standard model already captures relevant temporal dynamics through medication patterns themselves (e.g., polypharmacy indicating persistent treatment complexity).

B.3 Temporal Evolution of Medication Effects

Figure S1 (right panel) illustrates temporal evolution of medication predictor hazard ratios in the general time-varying model. Despite time-varying modeling not improving overall discrimination, interesting temporal patterns emerged for specific medication classes:

**Mood Stabilizers.** Strongest medication predictor showing persistent strong association across all time periods, with some attenuation over time. The consistently elevated hazard ratios reflect that mood stabilizers are rarely prescribed for true unipolar depression in clinical practice.

**Psychotropic Polypharmacy.** Total number of psychotropic classes showed relatively stable effects across time periods, suggesting polypharmacy reflects persistent treatment complexity rather than time-varying clinical features.

B.4 Machine Learning Feature Importance

Figure S3 (Panel B) shows XGBoost feature importance rankings for the general model. Medication variables dominated importance rankings, with mood stabilizers, psychotropic classes, and antipsychotics as the top three features. Clinical predictors maintained importance but were superseded by medication variables. This pattern confirms medications contain strong predictive signal while supporting the circularity interpretation: medications are powerful predictors precisely because prescribing reflects diagnostic suspicion.

B.5 Stability of Clinical Predictors

Importantly, when medications are included in the general model (Table S5), clinical predictors retain similar hazard ratios and significance levels. Psychotic features remain a strong clinical predictor, anxiety remains protective, and healthcare utilization patterns maintain similar associations. This stability across model specifications supports the validity of clinical model findings and confirms that medication inclusion does not fundamentally alter clinical predictor relationships.

B.6 Clinical Implementation Recommendations

Despite superior discrimination (C=0.874), we do not recommend the general model for clinical risk stratification for the following reasons:

- Medication patterns likely reflect diagnostic suspicion rather than independent risk factors
- Confounding by indication makes causal interpretation impossible
- Risk scores incorporating medications could inappropriately influence prescribing decisions
- Clinical model provides actionable predictions using only objective clinical features available at MDD presentation

The clinical model (apparent C=0.745, corrected C=0.720) achieves excellent discrimination without circularity concerns and is recommended for implementation in clinical practice. The general model serves as a valuable sensitivity analysis demonstrating robustness of findings and establishing the upper bound of predictive performance when all available information is utilized.
